# F-box receptor mediated control of substrate stability and subcellular location organizes cellular development of *Aspergillus nidulans*

**DOI:** 10.1371/journal.pgen.1010502

**Published:** 2022-12-12

**Authors:** Özlem Sarikaya Bayram, Özgür Bayram, Betim Karahoda, Cindy Meister, Anna M. Köhler, Sabine Thieme, Nadia Elramli, Dean Frawley, Jamie McGowan, David A. Fitzpatrick, Kerstin Schmitt, Leandro Jose de Assis, Oliver Valerius, Gustavo H. Goldman, Gerhard H. Braus

**Affiliations:** 1 Biology Department, Maynooth University, Maynooth, Co. Kildare, Ireland; 2 Department of Molecular Microbiology and Genetics and Göttingen Center for Molecular Biosciences (GZMB), Georg-August-Universität Göttingen, Göttingen, Germany; 3 Faculdade de Ciências Farmacêuticas de Ribeirão Preto, Universidade de São Paulo, São Paulo, Brazil; Oregon State University, UNITED STATES

## Abstract

Fungal growth and development are coordinated with specific secondary metabolism. This coordination requires 8 of 74 F-box proteins of the filamentous fungus *Aspergillus nidulans*. F-box proteins recognize primed substrates for ubiquitination by Skp1-Cul1-Fbx (SCF) E3 ubiquitin RING ligases and degradation by the 26S proteasome. 24 F-box proteins are found in the nuclear fraction as part of SCFs during vegetative growth. 43 F-box proteins interact with SCF proteins during growth, development or stress. 45 F-box proteins are associated with more than 700 proteins that have mainly regulatory roles. This corroborates that accurate surveillance of protein stability is prerequisite for organizing multicellular fungal development. Fbx23 combines subcellular location and protein stability control, illustrating the complexity of F-box mediated regulation during fungal development. Fbx23 interacts with epigenetic methyltransferase VipC which interacts with fungal NF-κB-like velvet domain regulator VeA that coordinates fungal development with secondary metabolism. Fbx23 prevents nuclear accumulation of methyltransferase VipC during early development. These results suggest that in addition to their role in protein degradation, F-box proteins also control subcellular accumulations of key regulatory proteins for fungal development.

## Author summary

Protein degradation controls all vital processes in cells including but not limited to cell cycle, development and protein recycling. Specific E3 ligase enzymes play important roles in protein degradation by labeling target substrates with ubiquitin. F-box proteins have been at the center of attention as subunits of E3 ligases, which determine substrate specificity. F-box proteins interact with the potential substrate causing their ubiquitination. The model fungus *Aspergillus nidulans* genome encodes a similar number of F-box proteins as humans. Development of *A*. *nidulans* is coordinated with production of bioactive secondary metabolites. F-box proteins have been shown to control various important processes in different organisms, but functions of total F-box proteins in a single organism have not yet been studied. We identified 74 *fbx* genes in the filamentous fungus. Numerous resulting F-box proteins are found in the nuclear fraction and interact with regulators. F-box proteins control vital processes such as stress responses, mitosis (asexual sporulation), meiosis (sexual fruit body formation) and secondary metabolite production. A particular example is Fbx23 which regulates development by sequestering inhibitory epigenetic methyltransferase VipC from the nuclear fraction where VipC prevents nuclear accumulation of NF-κB-like velvet domain regulator VeA.

## Introduction

Filamentous fungi are serious threat for food supply and safety spoiling about 30% of world harvest of plant-based products. Fungal infections threaten the health of more than 1.2 billion people resulting in 1.5 million mortalities every year [1]. The large group of more than 300 Aspergilli includes plant pathogen and aflatoxin producer *Aspergillus flavus*, the opportunistic human pathogen *A*. *fumigatus* and *A*. *nidulans* as reference organism. Understanding molecular mechanisms of fungal growth, development and secondary metabolite (SM) production helps to combat these organisms.

*A*. *nidulans* is amenable to study the transition from vegetative growth to development, which is connected to SM formation [2–4]. Germination of a mitotic conidium or a meiotic ascospore leads to long vegetative hyphal filaments, which differentiate upon reception of environmental signals. Light triggers formation of mitotic conidia via receptors [5]. Sexual fruit bodies containing ascospores are produced in soil as overwintering structures and are protected by bioactive SMs [6,7, 54]. *A*. *nidulans* development and SM formation are coordinated by transcriptional and epigenetic regulators such as the heterotrimeric VelB-VeA-LaeA complex [8].

Fungal development requires the balance between protein synthesis and selective protein turnover by the ubiquitin proteasome system (UPS) [9,10]. Central components of the UPS or most proteasomal subunits are essential for growth of *A*. *nidulans* [11,12]. About 20% of all *A*. *nidulans* proteins are ubiquitinated during hyphal growth and are located in the nucleus, whereas ubiquitinated proteins of single cell *Saccharomyces cerevisiae* are mainly in plasma membranes [13,14].

Target proteins are labelled by ubiquitin (Ub) covalently attached in three sequential steps by Ub activating (E1), conjugating (E2) and ligase (E3) enzymes. E3 ligases determine degradation specificity with cullin ring ligases (CRL) as the most diverse class. CRLs include SCF ligases with S-phase kinase-associated protein 1 (Skp1), Cullin 1 (Cul1) and F-box domain protein subunits [15,16]. Cul1 recruits Skp1 and the small RING protein Rbx1. Substrates are incorporated into SCF complexes by exchangeable F-box substrate receptors, which interact with Skp1 through their N-terminal 40–50 aa F-box domain [15]. The C-termini of F-box proteins bear different trp-asp (WD40) or leu rich repeats (LRR) to recognize and interact with specific substrates [17]. CRLs are activated by a change in conformation caused by covalent attachment of the ubiquitin family protein Nedd8 (neural precursor cell expressed, developmentally downregulated 8) to a conserved lysine of Cul1 [18,19].

Rapid F-box exchanges at SCF complexes are required during fungal development or adaptation to changing environmental conditions in order to degrade unnecessary proteins. Exchange cycles are self-controlled by the absence of target proteins, because the COP9 signalosome (constitutive photomorphogenesis 9, CSN) deneddylase recognizes CRLs, which are not interacting with substrates [20]. CSN mediated removal of Nedd8 (deneddylation) deactivates CRLs, resulting together with the F-box exchange factor Cand1 (cullin associated Nedd8 dissociated), in disassembly and then in SCF re-assembly [19,21,22]. CSN is essential in higher eukaryotes [23]. Dysfunction of *A*. *nidulans* CSN or Cand1 leads to block of sexual development, impaired light control and brownish colonies with accumulated secondary metabolites [24–28]. Fungal CSN interacts with the second deneddylase DenA, which supports conidiation [29].

Fungal F-box proteins, which had originally been primarily studied in yeasts, revealed multiple important functions removal or inactivation of proteins to support nutrition, growth, the circadian clock and development of multiple filamentous fungi [30,31] The genome of *A*. *nidulans* encompasses similar to humans approximately 70 mostly unknown F-box proteins [32], whereas *S*. *cerevisiae* has 21 and plants have more than 800 F-box proteins [33,34]. F-box mediated ubiquitination controls numerous processes including cell cycle, signaling, cancer or the circadian clock [34–36]. Several F-box proteins are involved in fungal development [30,31,37–39]. *A*. *nidulans* Fbx15 is required for asexual and sexual, and Fbx23 for light-dependent development [21,40]. In addition, Fbx23 is controlling together with Fbx47 CreA-mediated catabolite repression of *A*. *nidulans* [41]. Fwd1 of *Neurospora crassa* corresponding to Fbx23 targets the key regulator of the circadian clock Frq for proteasomal degradation [42]. Fbx15 of the human pathogen *A*. *fumigatus* controls nuclear location of the SsnF co-repressor for formation of gliotoxin and virulence [43]. Fbx50 (GrrA: glucose repression-resistant A) is required for maturation of *A*. *nidulans* ascospores [44].

A comprehensive analysis of all 74 F-box proteins from *A*. *nidulans* was performed to explore their molecular and developmental functions. A broad overview of putative Fbx substrates were identified from LCMS analysis of F-box-GFP pulldown samples from different fungal growth and stress conditions. A major group of F-box interactions with other proteins is nuclear. A more detailed analysis revealed that Fbx23 combines subcellular location with protein stability control in the nucleus to proceed and organize fungal development.

## Results

### SCF subunits SkpA and CulA interact with 46 fungal F-box proteins during growth, stress or development

**Fig 1:**
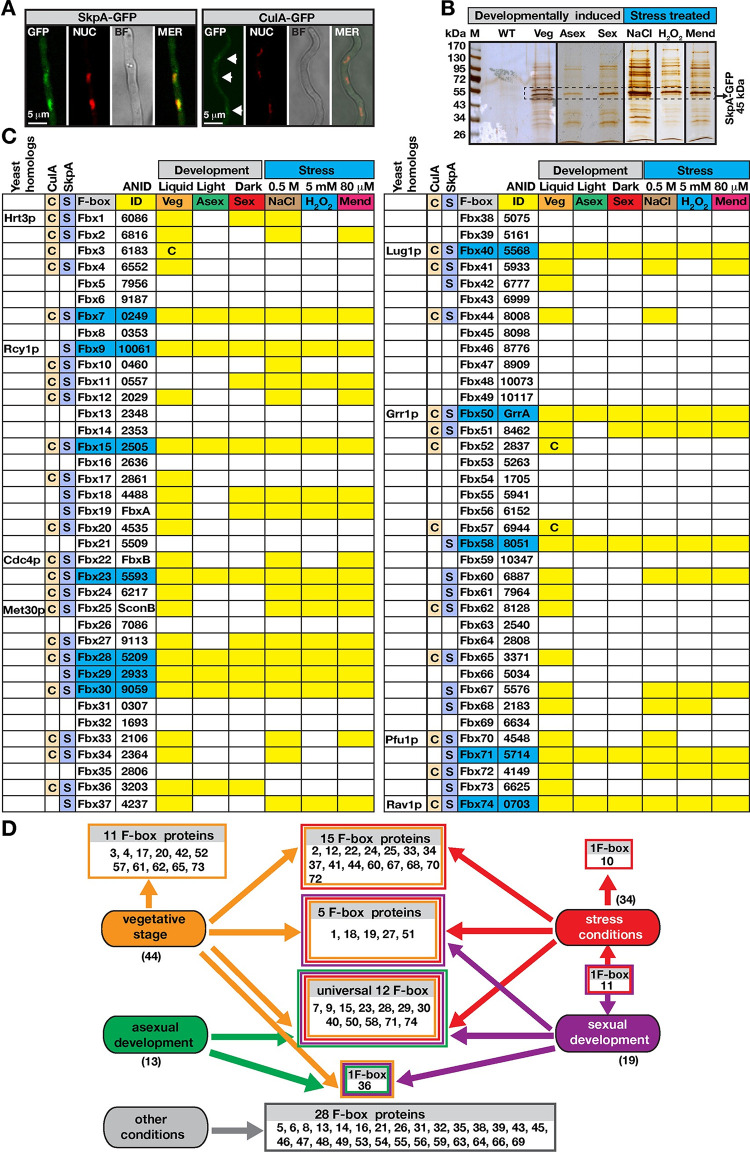
SCF complexes under developmental or stress inductions. (A) Cellular localization of SkpA–or CulA–GFP fusions during vegetative growth, which localized in the nucleus (arrows). Red DRAQ5 dye stains the nuclei. (B) Silver stained 10% SDS–PAGE of GFP trap for SkpA–GFP grown under different developmental (vegetative, submerged 24h, asexual under light 24h, sexual in the dark 24h) and stress conditions (5 mM H_2_O_2_, 80 μM Menadione, 0.5 M NaCl). Arrows indicate SkpA–GFP fusion (45 kDa). (C) Identifications of F–box proteins interacting with CulA or SkpA from (B) (S1–S9 Table). F–box proteins (Fbx) were numbered from Fbx1 to Fbx74, ANID indicates the corresponding locus numbers. Eight yeast homologs are given in first column. 46 of 74 F–box proteins are associated with SCF. Second and third columns represent CulA (highlighted by C) and SkpA (highlighted by S) pull–downs. The latter C indicates F–box proteins that were only found in CulA pull–downs from vegetative growth. Yellow shades indicate detection of corresponding F–box proteins and blue shading refers to an F–box protein present in all conditions. (D) A summary of SkpA–F–box interactions during development and stress. Colored of rectangles indicate either developmental stage or stress conditions. Universal F–box proteins are found to be associated with SkpA under all tested conditions.

Fungal growth, stress and development induce rapid responses to environmental signals that require selective protein turnover promoted by different SCF complexes. Endogenously expressed SkpA-GFP in its native locus was predominantly localized in the nucleus, whereas less endogenously expressed CulA-GFP was present there (Fig 1A). In several previous studies, we had shown that SkpA was expressed equally throughout all developmental stages [45,46]. Using SkpA-GFP fusion, SkpA interactors were identified by GFP pull-downs and mass spectrometry (MS) during vegetative growth, osmotic and oxidative stress, and asexual or sexual development (Fig 1B, S1–S7 Tables and Table A in S9 Table). Furthermore, using CulA-GFP fusion CulA interactors were identified from vegetative growth (Table B in S9 Table). For each condition, at least two independent biological replicates were used. Interaction partners of SkpA and CulA were filtered for unspecific peptides identified with free GFP control (S8 Table). SkpA and CulA mutually interacted in all purifications. 30 different F-box proteins associate with SkpA and CulA, 13 exclusively with SkpA and additional three only with CulA during at least one analyzed condition (Fig 1C). These 13 F-box proteins might also form heterodimers with CulA or SkpA without participating to SCFs. Besides bioinformatically identified F-box proteins (Fbx1 to Fbx70) [32], four novel F-box proteins (Fbx71-74) were discovered including Fbx74, which is an ortholog to yeast Rav1p, which harbors potential F-box motifs but is not classified as a *bona fide* F-box protein [47] (S1 Fig).

Only 12 of 46 interacting F-box proteins were universally found under all vegetative, stress and developmental conditions. They include developmentally required Fbx15, Fbx23 and Fbx50/GrrA. 44 F-box proteins of vegetative growth included an overlapping core of 32 F-box proteins present in at least one of the analyzed stress conditions. Fbx3/52/57 that interact only with CulA were exclusively observed during vegetative growth. Fbx36 was found throughout all developmental stages, but not during stress conditions. 19 F-box proteins were identified at sexual development, including 13 also present at asexual development. Fbx11 only appeared at sexual development and all stress conditions. Five F-box proteins (Fbx1/18/19/27/51) interacted exclusively during vegetative growth and sexual development (Fig 1C and 1D). 34 F-box proteins interacted with SkpA during oxidative stress and osmotic stress. 22 of them associated with SkpA for all three stress conditions. Fbx10 was exclusively found under osmotic stress and Fbx11 under all stresses as well as development, both interacted with SkpA and CulA.

The 46 SkpA/CulA F-box proteins can be assigned to a core of 32 (70%) interactions under vegetative growth and under one stress condition. This includes 12 (26%) general interactions found under all tested conditions. Additionally, 14 (30%) F-box proteins are condition-specific including 11 F-box proteins only found at vegetative growth, Fbx36 only at development, Fbx10 only in stress, and Fbx11 only in stress and sexual development. This implies that development and stress responses require oscillations in the composition of SCF complexes.

### Development requires 11% of *fbx* genes

Bioinformatic analysis suggests a potential for 28 additional F-box proteins in the *A*. *nidulans* genome which was not found to be interacting with SkpA and CulA in our study. Sequence and domain analyses supports a divergent evolution amongst them, where some candidates have only a partially conserved F-box domain (S1 and S2 Figs, S10 Table). Those F-box proteins with true F-box domain were named as high scoring F-box proteins. Low scoring F-box proteins do not possess a canonical F-box domain, but motifs like LRR and WD40 repeats that are often found in F-box proteins [32]. The 74 F-box proteins are distributed in four groups: (i) Three F-box proteins (Fbx1, Fbx50/GrrA, Fbx74) are shared by most eukaryotes including *A*. *nidulans*, human and plants. (ii) Fbx22/24/29 are shared between most fungi and higher eukaryotes. (iii) Fbx9 and Fbx25/SconB are fungal-specific. (iv) The largest F-box group is restricted to filamentous fungi, including Fbx15/23.

**Fig 2:**
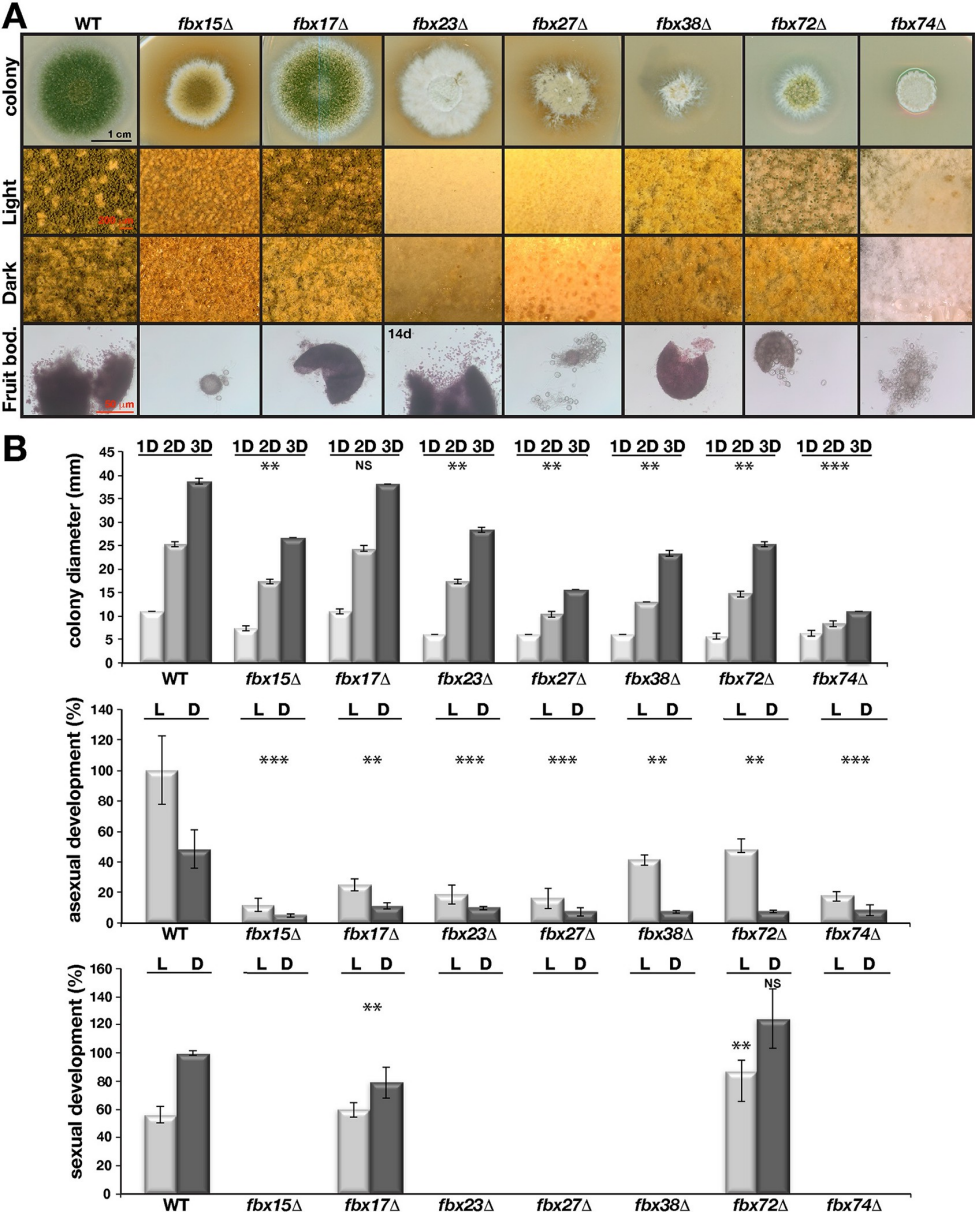
Requirement of seven *fbx* genes for light–dependent development. (A) The developmental responses of *fbx* mutants in comparison to WT strain. Close–up images (squares) of mutants with developmental defects. *fbx15*/*27*/*72*/*74* formed extremely small and *fbx17*/*23*/*38* generated several normal size fruit bodies devoid of ascospores. (B) Growth, asexual (mitotic) and sexual (meiotic) development of the mutants under light and dark conditions. Radial growth from 1, 2 and 3 days old cultures (mm). *fbx15*/*27*/*38*/*72*/*74* show slow growth. All mutants show reduced asexual sporulation. *fbx15*/*23*/*27*/*38*/*74* either does not produce fruit bodies or produce very few. Point inoculated strains (5x10^3^) were grown for 4 days for asexual and 5 days for sexual development, 3 days for radial growth at 37°C. Quantification of all parameters were performed from at least three different plates. Standard deviations are indicated. *** P≤0.0005, ** P≤0.005, *P≤0.05 values according to paired *t* test.

**Fig 3:**
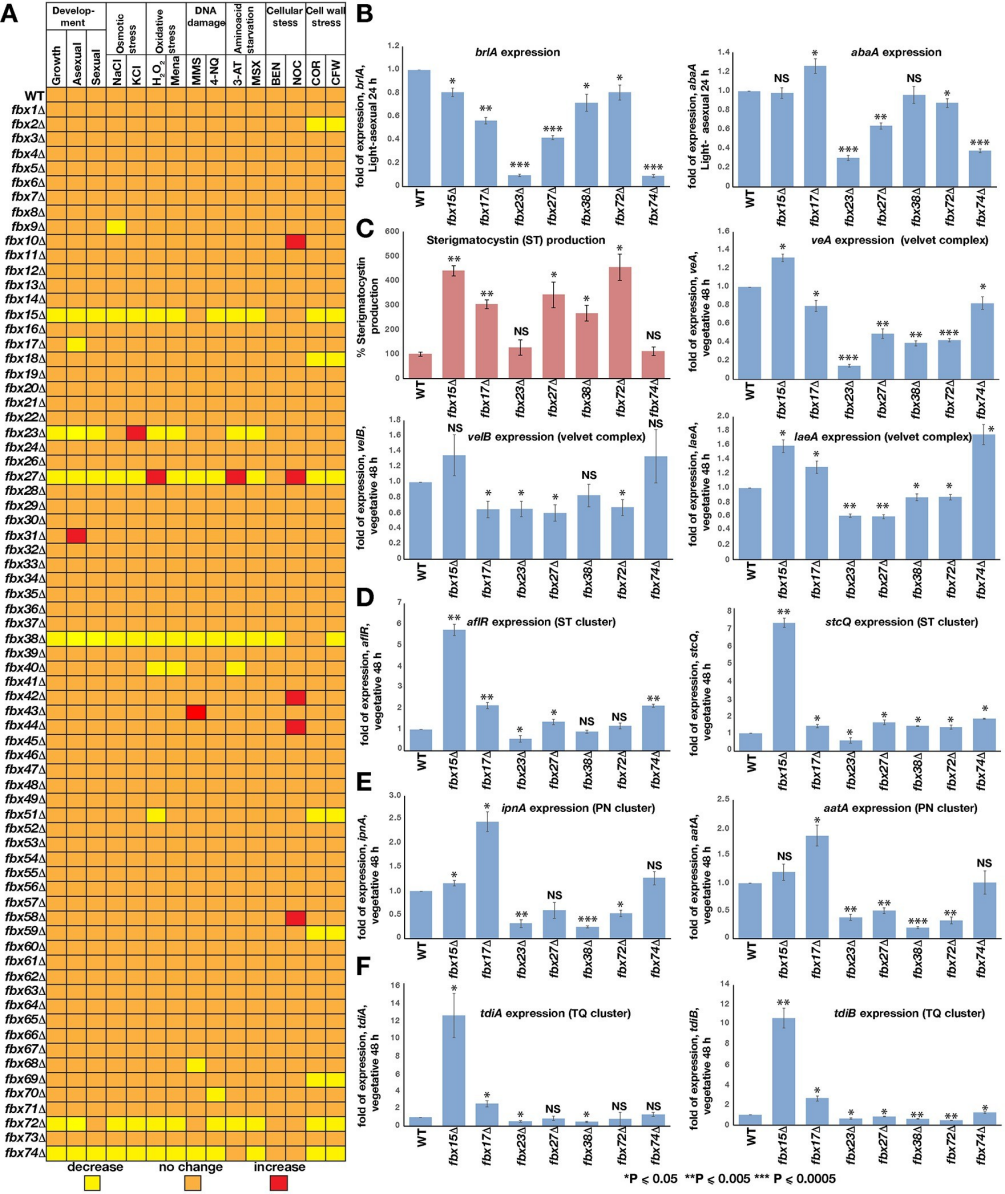
Control of developmental and SM gene expression by important F–box encoding genes. (A) *fbx* genes in development or stress responses. Heat map shows developmental patterns and stress responses of 74 *fbx* mutants (excluding previously characterized *fbx50*/*grrA*) in comparison to WT. *fbx15*/*27*/*38*/*72*/*74* mutants defective in development exhibit increased sensitivity to stress reagents. *fbx27Δ* is resistant to several stress factors. Several *fbx* mutants without developmental phenotypes show sensitivity to cell–wall stress, including *fbx2/18/51/59/69* (see also S8C Fig). 5x10^3^ spores were point inoculated for radial growth, on osmotic stress (1000 mM NaCl, 1000 mM KCl) and amino acid starvation MSX (2 mM). For the rest of the stress conditions 1x10^6^ spores were spread inoculated and paper discs soaked with 20 μl stress reagents (10% H_2_O_2_, 50 mM Menadione, 100 mM 3–AT, 10% MMS, 125 μg 4–NQ, 50 μg Benomyl and Nocodazole) were placed at the center of the plates and incubated for 3 days at 37°C. Diameter of the inhibition zones or radial growth was measured in three independent plates. (B) Expressions of *brlA* and *abaA* transcription factors in the *fbx15*/*17*/*23*/*27*/*38*/*72*/*74* in comparison to WT under light induced development for 24h. (C) Sterigmatocystin (ST) produced by developmentally defective *fbx* genes and expression of the heterotrimeric *veA*–*velB*–*laeA* genes at late vegetative phase (48h). ST levels increased in *fbx15*/*17*/*27*/*38/72*. ST was extracted from cultures grown on plates at 37°C for 5 days. (D) Expression of *aflR*, *stcQ* genes of ST cluster, (E) *ipnA*, *aatA* genes of PN cluster, (F) *tdiA*, *tdiB* genes of TQ cluster. qRT–PCRs come from two biological and one technical replicate. *** P≤0.0005, ** P≤0.005, *P≤0.05 values according to paired *t* test.

A systematic deletion of all *fbx* genes revealed that 73 *fbx* mutants were viable without altered germination efficiency, whereas *fbx25/sconB* encoding a sulphur metabolism regulator was essential (S3–S5 Figs). Previously, we had documented essentiality of *Aspergillus fumigatus fbx25* with a heterokaryon rescue experiment [43]. Mutant phenotypes were restored by reintroduction of GFP fusions of original genes (S6 Fig). Radial growth of six mutants (*fbx15*/*23*/*27*/*38*/*72*/*74*) was significantly diminished in glucose (Fig 2A and 2B). Their sexual development and asexual sporulation were blocked or reduced (Figs 2B and 3A). Growth of *fbx15*/*23/38/72* was restored by replacing glucose by alternative carbon sources (S7 Fig). Therefore, at least 11% (8 including essential *fbx25/sconB* and previously characterized *fbx50/grrA*) F-box proteins promote growth and development including 7 F-box proteins associated with SkpA at vegetative growth and additional one (*fbx38*) not found in the interactome (Fig 1).

Deletion of seven (10%) *fbx* genes displayed severe distinct developmental defects and are referred to as developmental F-box proteins. Asexual sporulation of *fbx15*/*17*/*23*/*27*/*38/72/74* was considerably decreased (Fig 2B). The *fbx15*/*27/74* mutants only produced small immature fruiting bodies without ascospores (Fig 2). *fbx17* mutant produced similar amounts of countable fruiting bodies of WT size and *fbx72* even larger but both without ascospores. *fbx23* and *fbx38* produced only few fruiting bodies. Longer incubation of *fbx23*/*38*/*72* led to very limited ascospore formation in media supplemented with extra uracil (Fig 2).

### F-box proteins control developmental and secondary metabolite gene expression

The impact of developmental F-box proteins on gene expression and SM production was analyzed. Expression of asexual regulatory genes *brlA* and *abaA* was reduced in *fbx23*/*27*/*74* mutants in comparison to WT (Fig 3B), in agreement with phenotypical data showing that these mutants present severely reduced asexual sporulation (Fig 2A). This correlation between reduced gene expression of *brlA* and *abaA* was not observed for *fbx15* mutant. Although expression of NF-κB-like velvet regulatory genes *veA*, *velB* and *laeA* for sexual development and SM revealed a complex regulation, general tendency was a down regulation of the velvet complex in mutants *fbx23*/*27*/*38/72* which have defects in sexual development. This downregulation was in agreement with the most of the sexual development defects of these strains except for *fbx15*/*74*. Absence of Fbx15 does not change *velB* and slightly increases *veA* and *laeA* expression. Fbx23 plays a prominent role and is required for expression of all three genes, especially *veA* (Fig 3C).

Developmental mutants are often impaired in sterigmatocystin (ST) production [6, 7]. Five *fbx* mutants (*fbx15*/*17/27*/*38*/*72*) produced significantly more ST than WT (Fig 3C). A comparison of genes involved in ST, penicillin (PN) and terrequinone (TQ) synthesis revealed 6 to 12-fold elevated expression of ST and TQ gene clusters in *fbx15* (Fig 3D–3F). *fbx* mutants showed increased expression of *aflR* regulatory and *stcQ* structural genes, except for *fbx23*. The PN cluster was downregulated in *fbx23*/*27*/*38*/*72*. Loss of *fbx17* increased PN cluster genes *ipnA* and *aatA* expression (Fig 3E). These results indicate that the majority of the *fbx* deficient mutant strains present a correlation between their phenotypes and relevant regulatory gene expression levels. This reflects a sophisticated positive and negative control of master regulators of fungal development and SM provided by seven developmental *fbx* genes.

### 20 F-box proteins are involved in various fungal stress responses

Five of seven developmental F-box proteins interacted with SkpA during stress. All *fbx* mutants were compared in a comprehensive stress analysis (Fig 3A). The majority of developmental *fbx* mutants were sensitive to most stress factors (S8 Fig), but some mutations provided specific stress resistance. *fbx27* deletion led to cytoskeleton, oxidative (S8A Fig) or amino acid starvation (3-AT) stress resistance. *fbx23* exhibited resistance to osmotic (S8B Fig), *fbx15* against cytoskeleton stress. *fbx72* and *74* did not cause sensitivity against DNA damage, amino acid starvation or cellular stress.

Nine additional mutants without obvious phenotype under normal growth conditions displayed stress sensitivity or resistance. *fbx2/18/51/59/69* mutants were sensitive to cell wall stress. *fbx10/42/44/58* mutants showed increased resistance to cellular stressors (S8C Fig). Therefore at least 16 (21.6%) including all developmental F-box proteins are involved in fungal responses against various stress signals.

### 24 F-box proteins are primarily nuclear and 38 are cytoplasmic during vegetative growth

**Fig 4:**
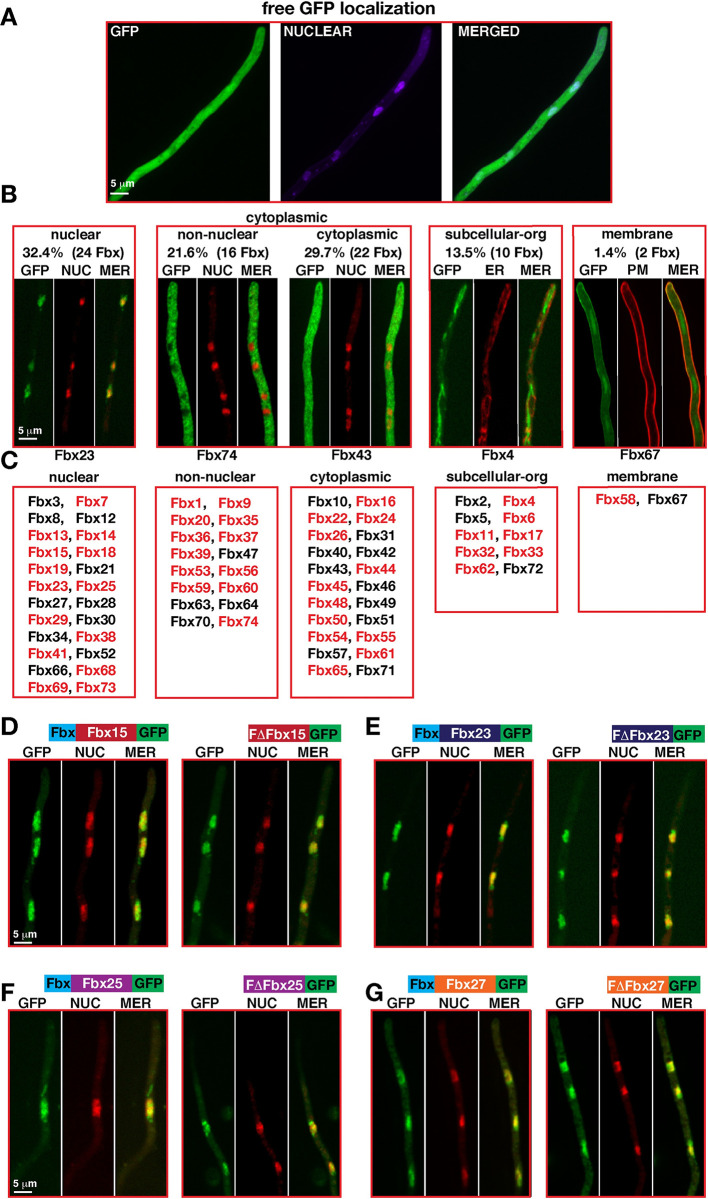
Subcellular localizations of Fbx–GFP during vegetative growth. (A) Subcellular localization of free GFP protein expressed under *gpdA* promoter. (B) Distribution of 74 Fbx–GFP (except for GrrA) expressed under *gpdA* promoter in four subcellular groups: (I) Nuclear F–box proteins 32.3%, (II) Cytoplasmic F–box proteins (IIa) 21.6% Non–Nuclear (nuclei are devoid of GFP), (Iib) 29.7% cytoplasmic (nuclei have GFP signal), III 13.5% subcellular organelles (cytoskeleton elements and secretion) (IV) 1.4% plasma membrane. Nuclei were stained red by DRAQ5, plasma membrane red by membrane dye FM4–64, and endoplasmic reticulum was labelled with SecA–mRFP fusion. Red marked F–box proteins contain a putative NLS signal. See (S9 Fig) for all localizations. (C) Representatives of F–box protein categories: nuclear Fbx23, non–nuclear Fbx74, cytoplasmic Fbx43, subcellular organelle or cytoskeleton Fbx4, plasma membrane Fbx67. Developmental Fbx15/23/27/38 are nuclear. (D–G) Subcellular localizations of Fbx15/23/25/27 and their F–box domain deleted forms (S9 and S10 Figs).

A substantial portion of eukaryotic proteasomes or SCF subunits are localized in the nuclei [48]. Our previous efforts to express a dozen of Fbx-GFP fusions under their native promoter failed to show localization patterns. Furthermore, pull-downs of these proteins also did not lead to identification of relevant F-box proteins in Mass Spectrometry (MS). Therefore, 73 Fbx-GFP fusions were expressed under constitutive glycolytic *gpdA* promoter as single copy in the biotin locus, which did not result in any overexpression phenotypes. Their cellular localizations along with the control strains expressing either free GFP and the control strain AGB551 without any GFP-tagged protein were determined during vegetative growth (Fig 4A–4C). Free GFP was mainly localized in the cytoplasm showing a homogenous distribution all over the fungal cells; (i) 32% (24/74) F-box proteins were primarily localized in the nuclei including half with predicted nuclear localization sequence (NLS, S1 Fig), and 10 with small cytoplasmic distributions (S9 Fig). Four developmental F-box proteins 15/23/27/38, essential Fbx25/SconB and Fbx19/FbxA were in this group; (ii) 51% (38/74) showed two distinct patterns of cytoplasmic localization with 22 primarily cytoplasmic F-box proteins not excluded from nuclei (cytoplasmic), and 16 (non-nuclear) F-box proteins completely excluded from nuclei, as developmentally relevant Fbx74 with three predicted NLS; (iii) 10 of the remaining 12 F-box proteins were associated with subcellular structures such as microfilaments or the endoplasmic reticulum (ER, S9 Fig) and included developmental Fbx17 and 27; and (iv) Fbx58 and Fbx67 are localized to plasma membrane. F-box domains of five developmental F-box proteins were not relevant for cellular localization, because F-box domain deletions did not change their localizations (Figs 4D–4G, S9 and S10). Developmental F-box proteins are distributed to nucleus, cytoplasm and subcellular structures, supporting that specific protein turnover at different cellular locations is important to initiate differentiation.

### 45 F-box proteins interact with 743 proteins, of which 224 are nuclear

**Fig 5:**
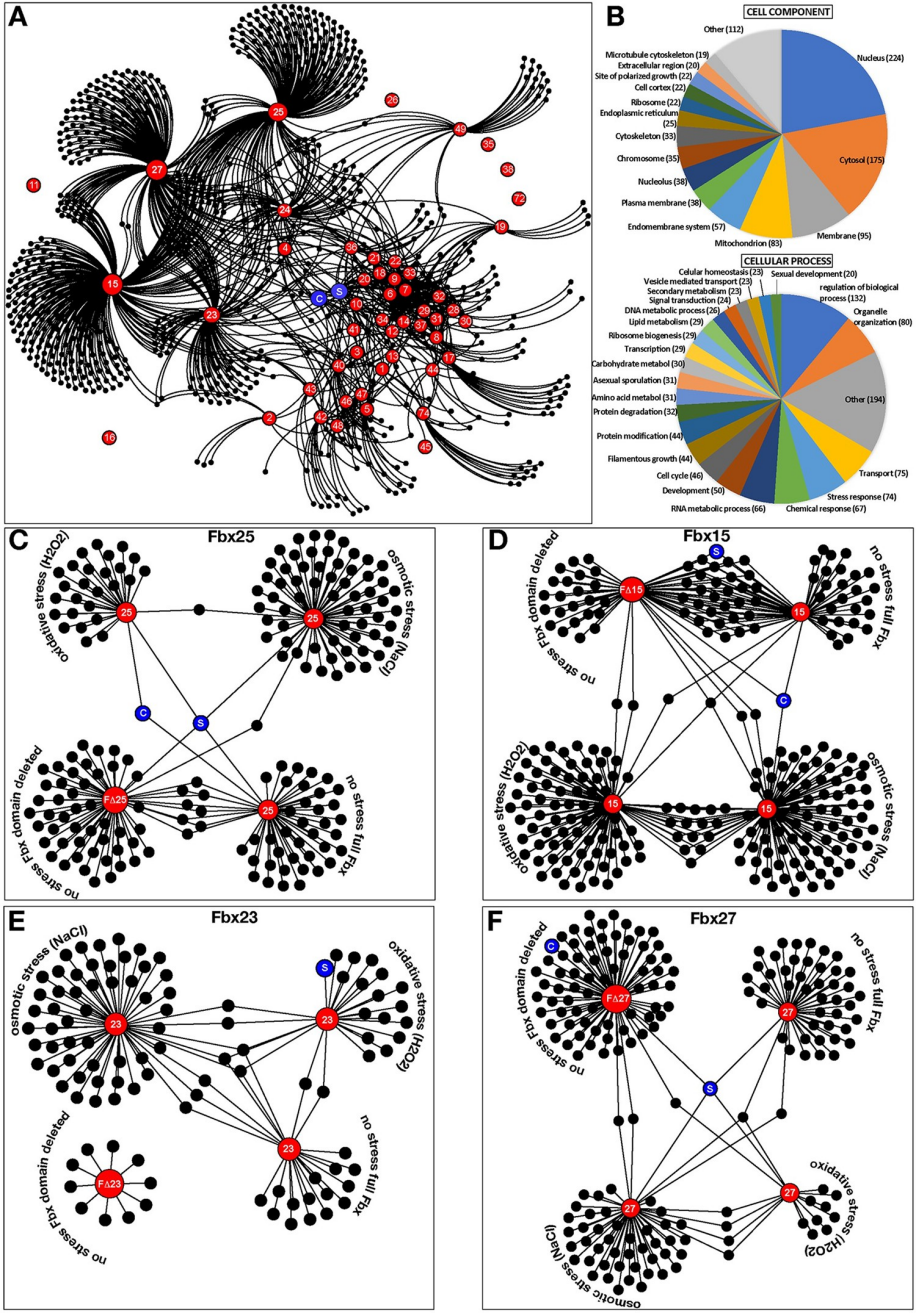
More than 700 cellular proteins are associated with 45 F–box proteins *in vivo*. (A) Interaction web of 743 proteins associated with 45 F–box proteins under vegetative conditions. Proteins were identified as a result of GFP trap of the Fbx–GFP fusions from vegetative cultures at 30°C for 24h. Purifications (S11 Fig: silver stained gels) were performed as two biological replicates. F–box proteins are given in red. Blue spheres C: CulA, S: SkpA. Black spheres are associated proteins. (B) F–box associated proteins are primarily nuclear and regulatory and are grouped into 20 categories based on cellular components and functions (see S13 Table). (C) A web of full length Fbx25 protein associations under normal, osmotic and oxidative stress conditions. Associations of Fbx25 without F–box domain (FΔFbx25). (D–F) Protein associations of Fbx15/23/27 and their F–box truncation under normal and stress conditions. Red spheres represent the corresponding F–box protein used in purification. Blue spheres C: CulA, S: SkpA.

Forty-five high scoring F-box proteins including developmental F-box proteins15/17/23/27/74 interact with 743 proteins (Fig 5A and 5B, S11–S14 Tables), including SkpA or CulA as common Fbx interactors. The major group of F-box interactors were nuclear (224/743), followed by 175 cytoplasmic proteins. Functional categories revealed mostly regulatory (132), organelle organization (80), transport (75), and stress (74) or chemical (74) response proteins associated with F-box proteins. Even F-box proteins without obvious phenotype interacted with regulatory proteins (S14 Table). This includes BimG phosphatase (anaphase) (Fbx5), MobB (polarity) and HapE (Fbx24), PkaC, MAPKK AnSte7 (Fbx43), HogA (Fbx47). F-box proteins interact with each other under specific conditions, as Fbx4 was in Fbx15/25/49 pull-downs or Fbx51 in Fbx15/23 pulldowns under osmotic stress. This suggests that Fbx4 is even a substrate of the other Fbx proteins.

### Essential Fbx25/SconB recruits metabolic and regulatory proteins

Fbx25/SconB interacted with 48 proteins of primary metabolism, putative DNA binding, chromatin control, TorA kinase and CasA caspase proteins (Fig 5C). Deletion of *torA* kinase in *A*. *nidulans* causes growth arrest with very short germlings [49]. Similarly, caspase gene deletions influence growth severely *in A*. *fumigatus* [50]. Osmotic stress led to 65 associated proteins including regulatory proteins such as phosducin-like C, fatty acid regulator FarA, DenA, SrpkG and Cla4-like kinase, or diterpene transcription factor (TF) PbcR. Oxidative stress caused association with 29 proteins as developmental TF NsdD and several SM proteins. Truncation of the F-box domain (FΔFbx25) increased the number to 58 associated proteins, with SkpA interaction as one of only eight shared proteins in comparison to intact Fbx25. FΔFbx25 interacted with the meiosis TF NosA or the HECT type Ub-ligase HulA, which was also interacting with Fbx18 (Fig 5C). Reduced SCF assembly of FΔFbx25 presumably stabilized interacting proteins.

### Developmental Fbx15 changes its interactome considerably under stress conditions

Fbx15 and FΔFbx15 each copurified 60 proteins (Fig 5D). Fbx15 interactions comprised several regulatory proteins such as MAPKK AnSte7, high mobility group C and Fbx4. Approximately half of the 60 proteins interacted with Fbx15 as well as FΔFbx15. Lack of F-box domain did not influence Fbx15 interaction with other SCF subunits (S12 and S13 Tables). Most of 94 proteins associated with Fbx15 during osmotic stress were not found under non-stress conditions. Regulatory proteins such as ScrC (suppressor of *crzA*), cell polarity SpaA, NudC, CmkD (stress kinase), Sds3 (deacetylase), HapC (CCAAT binding complex member), and proteins involved in biosynthesis of SMs such as ST (StcW, StcT, StcE) and TQ (TdiA, TdiC) interact with Fbx15 under osmotic stress.

Under oxidative stress conditions a reduced number of Fbx15 peptides were identified in comparison to no stress condition (18 vs 5). Similarly, oxidative stress also led to identification of less Fbx23 peptides (22 vs 13) or Fbx25 (33 vs 9). The number of Fbx15 associated proteins increased to 98 including 15 proteins shared during oxidative or osmotic stress. Interacting adenylate cyclase CyaA and associated CapA, septation regulators SepA and SepK, TorA, TFs SteA (yeast Ste12) and DevR, kinases Gcn2, Yak1 and Ste20 are potential links to the pleiotropic *fbx15*Δ phenotype.

### Developmental Fbx17/23/27/74 interactions include sets of RNA-associated, regulatory, signaling or redox balance proteins

Vegetative Fbx17 interacted with nucleotide and RNA metabolism, binding, modification, processing and ribosome biogenesis proteins. FΔFbx23 is less stable than Fbx23, resulting in only 11 protein interactions. Intact Fbx23 interacted with 24 proteins as TF SltA, LaeA-like methyltransferase LlmB (VipC) or TF Taf30 (Fig 5E, S11–S13 Tables). Osmotic and oxidative stress resulted in 58 and 24 associated proteins, respectively. TF SltA, zinc finger ZipC, CmkD were present under osmotic stress. SltA was also pulled down by Fbx23 under oxidative stress along with several SM proteins of the ST or TQ pathways. Fbx27 resulted in 47 associated proteins, including regulatory and signal transduction proteins (Fig 5F) such as serine/threonine kinase Atg1, hog pathway YpdA or kinase subunit PkaR.

Fbx27 and FΔFbx27 only shared SkpA as partner. 75 FΔFbx27 interacted with proteins comprise chaperonin complex Cct1, Cct7, heme biosynthetic enzyme HemC, cell wall antigen GfsA or chitin synthase ChsB. Fbx27 associated with 52 and 16 proteins under osmotic and oxidative stress, respectively. Both stress conditions shared only four proteins with HapC and ZipC being amongst the 52 Fbx27 interactors under osmotic stress. Fbx74 interacted with redox balance enzymes including a putative peroxiredoxin or three oxidoreductases, indicating a link to cellular redox balance (S11 Table). The F-box interactome revealed a dynamic protein environment with considerable changes upon external signals including control of numerous regulatory proteins for accurate fungal growth, development or stress response.

### Developmental Fbx23 prevents nuclear localization of methyltransferase LlmB/VipC, which results in stabilization of nuclear VeA during early development

**Fig 6:**
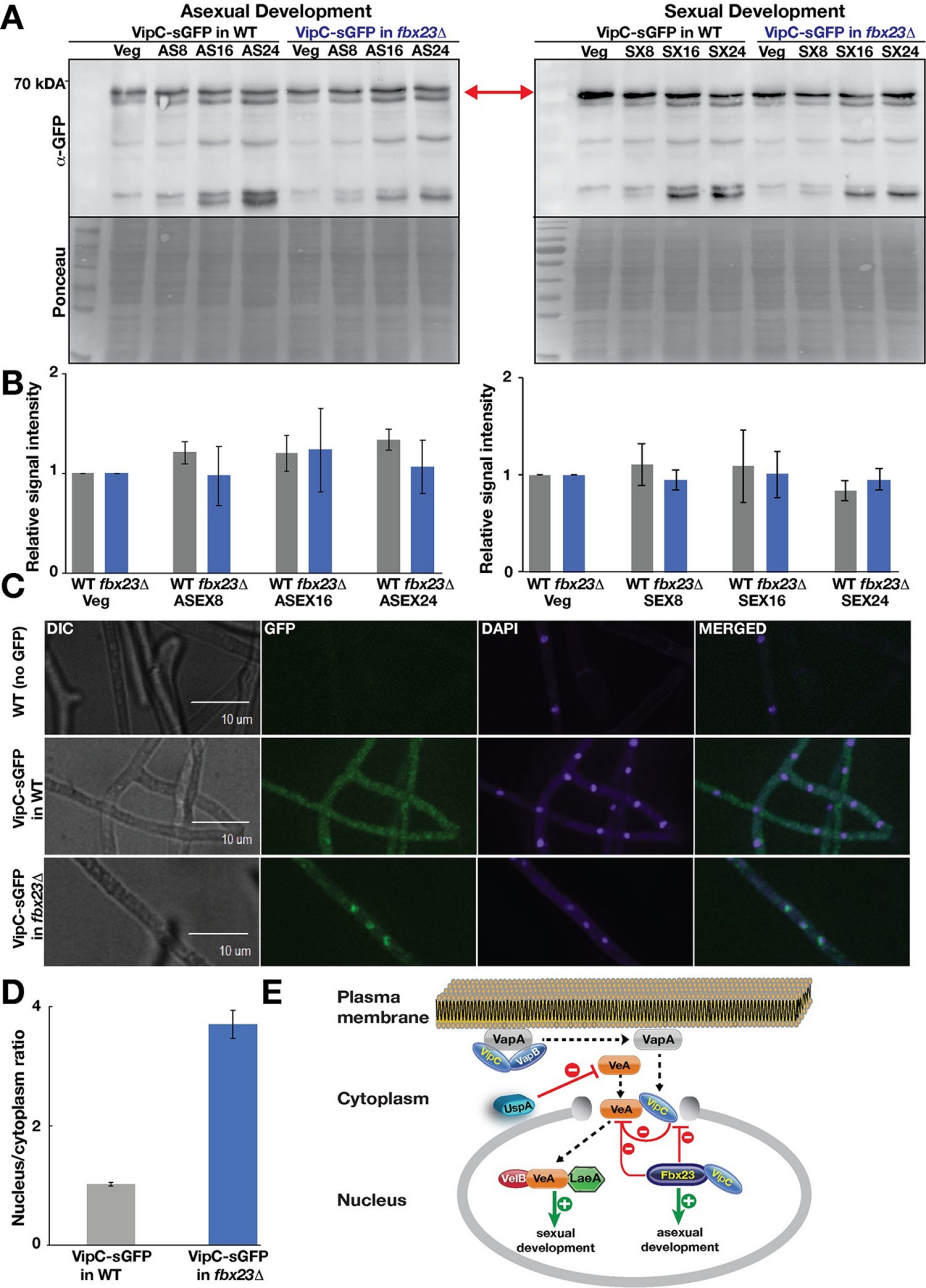
VipC–GFP nuclear accumulation is prevented by Fbx23. (A) VipC–GFP protein abundance is independent of Fbx23 during development of *A*. *nidulans*. Expression of VipC–GFP fusion during asexual and sexual development in WT and *fbx23*Δ strain detected by α–GFP antibody. Ponceau S staining serves as loading control. (B) The quantification of the VipC–GFP fusion signal is based on three independent biological replicates. Protein levels were normalized to Ponceau S staining of the blots. Error bars represent standard deviation. (C) Subcellular localization of VipC–GFP in WT and *fbx23*Δ strain grown for 20 h in light at 37°C. (D) Quantification of VipC–GFP fluorescent signals in the cytoplasm compared to the nucleus in WT and *fbx23*Δ performed by SlideBook 6.0 software package. For *vipC*:*gfp* in Δ*fbx23* the mean of 54 ratios was calculated, whereby for *vipC*:*gfp* in wild type 43 ratios were considered. Error bars represent the standard error of the mean from two biological replicates. (E) VipC is bound to the plasma membrane protein VapA under normal growth conditions and is released by external signals to enter the nucleus. Fbx23 primarily localizes to nuclei and prevents VipC but not VeA nuclear localization. VipC was shown to affect VeA protein stability in the nucleus [46]. Dashed lines indicate transport of the proteins. Green lines indicate positive/promoting effects and red lines indicate inhibiting/preventing coherences.

Fbx23-GFP associated with the methyltransferase LlmB (VipC) during vegetative growth (S11 Table) suggesting that Fbx23 affects VipC stability. VipC is part of an epigenetic methyltransferase signal transduction pathway from plasma membrane to nucleus. VipC reduces stability of the NF-κB-like velvet domain regulator VeA together with the deubiquitinase UspA during development and interacts with VeA in the nucleus [46, 51]. Functional VipC-GFP protein levels were stable during vegetative growth and up to 24h in either asexual or sexual development inducing conditions. VipC stability was independent of Fbx23 (Fig 6A and 6B). UspA and VipC reduce VeA protein amounts during later developmental time points [51].

Under non-stress WT conditions, VipC is equally distributed through the hyphae including cytoplasm and nuclei (Fig 6C) and can accumulate at plasma membrane spots linked to the membrane-bound protein VapA [46]. VipC is released by yet undefined external signals and is able to enter the nucleus and promote asexual development [46]. VipC-GFP nuclear accumulation increased more than three-fold in Δ*fbx23* compared to WT (Fig 6C and 6D). VeA localizes to nuclei independently of Fbx23 (S12 Fig.). This supports a complex interplay where Fbx23 impairs the VipC nuclear import and also reduces the nuclear VeA population as described earlier [46].

## Discussion

F-box proteins represent a large group of receptors of CRLs for specific ubiquitin labelling of substrates. More than 600 human CRLs are assembled on a set of eight different cullins, including three which are conserved in fungi [52]. Human CRL1 corresponds to fungal CulA as conserved scaffold for SCFs representing the largest group of eukaryotic CRLs. Human and *A*. *nidulans* genomes encompass a similar number of different F-box proteins [39]. All 74 F-box encoding genes of the mold *A*. *nidulans* were compared for their contribution to growth, development and stress response. Only the fungal-specific *fbx25/sconB* gene was essential for growth, whereas deletion of 73 F-box proteins resulted in viable strains. 52 *fbx* mutants revealed at least one phenotype or protein interaction or combinations of both: 43 F-box proteins were part of the SkpA/CulA interactome and the others affected growth, development or provided specific stress resistance or sensitivity (Fig 3). F-box proteins have been intensively studied in unicellular fungi as the budding yeast *S*. *cerevisiae*. Yeast has much less F-box proteins than *A*. *nidulans*. This might be the reason why as example the deletion of *fbx22* has no severe effect in *A*. *nidulans*, whereas yeast Cdc4 (Fbx22) has been assigned with diverse functions [30,31].

Our data suggest that *A*. *nidulans* produces already during vegetative growth numerous F-box proteins required for stress. This supports that the fungus is prepared to respond to a changing environment, which includes abiotic as well as biotic factors as other organisms including bacteria [53] or fungivorous animals [54] as they are present in the soil microbiome. We assume that most of the 74 F-box proteins might fulfil specialized rather than shared or redundant functions to be prepared to different complex communities.

8 F-box proteins positively affect fungal growth, whereas 16 *fbx* mutants exhibited specific stress resistance or sensitivity. Developmental F-box proteins affected expression of regulators for differentiation and SM. Fungal-specific Fbx15 is involved in fruit body formation, various stress responses and the repression of mycotoxin ST presumably by degrading the AflR transcription factor. F-box proteins might act as repressors of sterigmatocystin production by potentially degrading regulatory proteins or structural proteins of this pathway. *A*. *fumigatus* Fbx15, which is also required for stress, represses gliotoxin synthesis and is essential for pathogenicity [43]. Therefore, we suggest Fbx15 to be a general repressor for several metabolites and a comprehensive metabolite analysis of the *fbx15* deletion strain would be an interesting future project.

62% of F-box proteins carry conventional NLS sequences, but there are nuclear F-box proteins without NLS and non-nuclear F-box proteins with NLS. These proteins might be imported into the nucleus under different conditions or can be co-transported by the NLS of interacting proteins, which remains to be proven in future studies. The 26S proteasome is enriched in the nucleus [48] suggesting that a significant number of proteins are modified and degraded in the nuclear fraction. Consistently, the number of nuclear F-box-associated proteins is highest. A similar analysis for other organisms is yet to be undertaken. Analysis of 17 *Arabidopsis* F-box proteins revealed two nuclear and six nucleo-cytoplasmic proteins [55] suggesting possible links between location, function and stability. Fbx58 and 67 are both at plasma membrane and nucleus. The fungal VipC-VapA-VapB methyltransferase complex shuttles from plasma membrane to nucleus to transmit a signal to control gene expression in *A*. *nidulans* [46]. *A*. *fumigatus* Fbx15 shuttles in complex with SsnF between cytoplasm and nucleus [43]. F-box proteins might shuttle from membrane into the nucleus and back under specific conditions to transmit signals by ubiquitination of target proteins.

This study provides a set of putative interaction partners of F-box proteins from GFP-trap couples LCMS analysis. These data can be used in follow-up studies by using two-tagged proteins expressed under their native promoter to mimic a more biological relevant situation. This pilot mass-pull-downs were conducted with the *gpdA* promoter controlled F-box protein encoding genes, which did not show any overexpression phenotype, probably due to the high F-box-protein turnover. The interactome of 45 F-box proteins and more than 700 interaction partners at vegetative growth included 30% primarily nuclear proteins. The majority of interactors are regulatory proteins like kinases, transcription factors, chromatin control, DNA repair and cell cycle proteins (S14 Table). Potential target pathways might be controlled by F-box mediated ubiquitination including pheromone response, target of rapamycin (mTOR), protein kinase A and C and high osmolarity glycerol kinases. The UPS mainly downregulates signal transduction including the mammalian DEPTOR protein as an inhibitor of mTOR, which is targeted by SCF^ßTrCP^ E3 ligase to control survival and autophagy. mTOR is degraded by FBXW7 and several MAPKs are targets of F-box proteins [56, 57]. Cell cycle, DNA repair and meiotic transcription factors are linked with F-box proteins and several anaphase promoting complex (APC) subunits, which connect the two E3 Ub-ligases APC and SCF. Both primary and SM regulatory proteins specifically interact to F-box proteins, which therefore organize both biochemical networks.

46 *A*. *nidulans* F-box proteins interacted with SkpA and/or CulA during either vegetative growth, development or at least one of three tested osmotic or oxidative stress conditions. The filament is well prepared for stress protection, because almost all (44) F-box-SkpA and F-box-CulA interactions are already present at vegetative growth. The ratio of 43 F-box-SkpA interactions (43 out of 74) is similar to the corresponding ratios of budding (13 out of 21) or fission yeast (11 out of 20) [58,59]. Half of the carbohydrate binding Arabidopsis F-box proteins were associated with at least one Skp1-like ASK[60]. In humans 33 out of 69 F-box proteins are associated with human SKP1 (http://www.uniprot.org/uniprot)).

The conventional hypothesis proposes that diversity of SCF complexes depends on F-box-SkpA/1 binding of interchangeable F-box proteins [61]. However, fungal Fbx3/52/57 and human Fbxl21, Fbxl3, FBO4 and FBXW11 interacted with CulA/CRL1, but not with SkpA/1. Developmental Fbx15/25/27 interact with SkpA with or without F-box domain, and FΔFbx15, FΔFbx27 interacted in addition with CulA. This suggests additional domains of F-box proteins, which interact with SkpA and/or CulA for SCF incorporation. Alternative SkpA/Cullin interaction motives are an interesting topic for future studies. These interactions could also represent the degradation process of FΔF-box proteins, which are indirectly associated to CRLs. FΔFbx23 was less stable than intact Fbx23. Some F-box proteins can interact with each other to mediate their degradation. A Skp1-free non-canonical Fbx degradation pathway for yeast Met30 (Fbx25/SconB) is important for chromosome stability and protection against heavy metal stress[62]. This supports a complex interplay of potential F-box interaction domains: (i) Conventional F-box of SCFs as SkpA dependent destruction box for self-destruction or degradation of targets due to (ii) additional surfaces to recruit one or several substrates. (iii) F-box is used for non-SCF F-box-Skp1 interactions as V-ATPase or kinetochore assembly [47]. (iv) F-box proteins comprise additional interaction domains for either SkpA and/or CulA located outside of F-box domain. F-box evolution requires only the addition of an F-box domain to any protein to rapidly introduce additional layers of protein function control and to further destabilize not only this protein itself but also its interacting proteins. The fact that FΔFbx25 version of essential Fbx25/SconB is viable corroborates the existence of diverse functions of F-box proteins besides targeting substrates for ubiquitination through their F-box domain.

Most vegetative and stress interactions correlate suggesting vegetative protective anticipation of stress as described for human fungal pathogens [63]. Fbx36 is present during growth and development without stress. Only Fbx10 interacts stress-specific and Fbx11 during sexual development and stress. The 19 sexual interactions include all asexual interactions corroborating tight relationships between both programs. This is further supported by developmental Fbx23 which exhibits a complex stability control at the interface between asexual and sexual development and is involved in metabolic control [41,46,51]. A connection of Fbx23 to circadian clock regulator Frq, as it was shown in *N*. *crassa* [42], was not detected in *A*. *nidulans* with the experimental setup used in this study. Deubiquitinating UspA destabilizes the NF-κB-like velvet domain regulator VeA. VeA is required for the induction of sexual fruiting body formation. VipC supports asexual development, because it reduces the nuclear VeA pool [46] and impairs nuclear VeA localization. VeA-GFP localizes independently of Fbx23 to the nucleus (S12 Fig.). Fbx23 interacts with VipC, but does not reduce its protein levels. Instead Fbx23 excludes VipC which interacts with VeA from the nucleus and changes its subcellular localization. Fbx23 does not affect VeA nuclear import but indirectly affects its stability, by reducing nuclear location of VipC (Fig 6E). Dashed lines indicate transport of the proteins. Green lines indicate positive/promoting effects and red lines indicate inhibiting/preventing coherences. The complex interplay between VeA stability and nuclear location of the methyltransferase VipC by Fbx23, and by the deubiquitinase UspA illustrates the interconnected turnover of regulatory proteins to organize fungal differentiation programs. This first systematic analysis of *A*. *nidulans* F-box proteins allows to elucidate putative substrates and individual F-box functions besides being adaptors for SCF complexes and emphasizes their importance in regulation of fungal growth, development, secondary metabolite production and pre-emptive stress protection.

This study provides numerous data on *fbx* protein deficient fungal mutant strains, their phenotypes, stress responses, their localizations and potential cellular interaction partners. This serves as base for follow-up studies for the detailed molecular mechanisms how F-box proteins are involved in various fungal stress responses. The potential 700 interaction partners of the F-box proteins have to be further verified by approaches such as co-immunoprecipitation, yeast two hybrid, and biomolecular fluorescence complementation. This will be an interesting starting point for exploring F-box protein functions in a multicellular fungus.

## Materials and methods

### Strains, plasmids, oligonucleotides and cultivation of microorganisms

Fungal strains are listed in S15 Table, deletion cassettes in S16 Table, oligonucleotides in S17 Table, GFP fusions in S18 Table and plasmids are listed in S19 Table. *A*. *nidulans* AGB551 (*veA*+) served as WT recipient for transformations of 73 *fbx* deletion cassettes and the resulting set of deletion strains were screened in a pilot study for involvement in CreA-mediated catabolite repression (63). Localization and interactome studies were performed with mutants transformed with the respective *fbx-sgfp* fusion. Only *fbx25-sgfp* and *f*Δ*fbx25-gfp* fusions were transformed into a WT strain. Unless otherwise stated, fungi were grown in minimal medium containing glucose as carbon source (GMM) supplemented with vitamins or 2% agarose for solid medium at 37°C. In carbon source experiments, glucose was substituted by an alternative carbon source as indicated in corresponding Figs.

### Stress tests

In stress tests, stress reagents were either added to GMM or were given on the paper discs. For paper discs experiments, fresh fungal spores (1x10^6^) were spread on plates and paper discs with stress reagent was placed on the middle of the plate and the strains were grown for 2 days at 37°C. For other stressors added to GMM, (5x10^3^) fungal spores were point inoculated and grown for 3 to 4 days at 37°C. Following concentrations of stress agents were used. Osmotic stressors were added to medium at following final concentrations: 1000 mM NaCl; 1000 mM KCl, oxidative stressors were added on paper discs at following concentrations: 10% H_2_O_2_; 50 mM Menadione. DNA damage stressors were added to paper discs, 10% Methyl methanesulfonate and 125 μg 4-Nitroquinoline 1-oxide. Amino acid starvation agents were added on either GMM plates (2 mM Methionine sulfoximine) or paper discs (4 mM 3-Amino triazole). Both cellular cytoskeleton stress agents were added on paper discs, each 50 μg, Nocodazole and Benomyl. Cell wall stressors, 40 μg Congo red and 20 μg Calcofluor white were added to GMM at final concentrations.

### GFP trap protocol and LC-MS protein identification

Immunoprecipitation (IP) of SkpA, CulA and 49 Fbx-GFP fusions employed GFP-TRAP sepharose or magnetic particles (Chromotek) with minor modifications (S1 Text) as described [64].

### Confocal microscopy

Strains expressing SkpA, CulA, Fbx-GFP fusions were grown in liquid GMM in 8-well borosilicate cover glass chambers at 37°C for 16 hours (Nunc). Confocal images were recorded as given in detail [64]. Further details of confocal microscopy is available in S1 Text.

### Bioinformatics, statistical analysis

Fbx domains were searched by using online tools, including NCBI protein blast tool (https://blast.ncbi.nlm.nih.gov/Blast.cgi), EMBL-EBI protein sequence and analysis tool (http://www.ebi.ac.uk/interpro/), and NLS mapper (http://nls-mapper.iab.keio.ac.jp/cgi-bin/NLS_Mapper_form.cgi) by using cut-off score 4.0. Statistical significance of the quantifications was evaluated by using online (http://www.graphpad.com/quickcalcs/) paired *t* test. Each developmental quantification was performed as at least three biological replicates.

### Secondary metabolite analysis

For the procedures of fungal SM extraction see S1 Text.

## Supporting information

S1 Fig Domain architectures of the putative 74 F-box domain proteins from *Aspergillus nidulans*. Top scale indicates the sizes of the proteins in amino acids (from 0 to 1000). Proteins exceeding the maximum length scale are Fbx11, 22, 73 and 74. Locus identities are given on the left-hand side as AN-numbers. Yeast homologs are given on the right-hand side. Identified F-box domains are indicated as green squares, common motifs such as Leucine Rich Repeats (LRR) or WD-40 domains found in F-box proteins are shown as blue triangles and red oval spheres, respectively. Red star represents Nuclear Localization Signal (NLS). Brown hexagons represent Ankyrin Repeats (AR). Polypeptide-transport-associated (POTRA), Transmembrane Helix (TM-Helix), Regulator of the ATPase of vacuolar and endosomal membranes (RAVE). Rest of the descriptions of the further identified domains present on F-box proteins are given at the bottom of the Figure.
(TIF)

S2 Fig Orthologs of 74 F-box domain proteins from yeast, fungi to human. Orthologs of 74 F-box domain proteins were determined from single-celled yeasts, filamentous fungi to human using a reciprocal best BLAST hits strategy. *A*. *nidulans* F-box proteins have more common orthologs in filamentous fungi, and less in single-celled yeast and human. Accession numbers or locus ID were given in S10 Table.
(TIF)

S3 Fig Confirmation of the *fbx* gene deletion events by the Southern hybridization. (A) An illustrative depiction of the deletion events via homologous gene replacement. 5´ UTR: five prime untranslated region, 3´ UTR: three prime untranslated region. Dashed lines with shadows represent either 5´ UTR or 3´ UTR hybridizing Southern probes (ranging from 500 to 800 bps). *Aspergillus fumigatus*-derived *pyrG* gene, *AfpyrG* served as a selection marker for the deletion events. (B) Southern hybridization results of *73 fbx* gene deletion events. The *fbx* genes were given at the top of the blots, below of which restriction enzymes used for controlling each deletion event and Southern probes (5’ or 3’ UTR) were highlighted. Only *fbx25* (encoding SconB) was lethal (double band one ectopic deletion cassette and one endogenous *fbx25* locus). Sizes of the bands are given at the bottom of the blots in kbps. W: WT locus, Δ: respective *fbx* deletion.
(TIF)

S4 Fig Confirmation of locus specific integration of *fbx*::*gfp* fusions into biotin locus. (A) Schematic representation of *fbx*::*gfp* cassettes, original biotin locus (*biA*) in WT and *fbx*Δ strains, and biotin locus in complementation strains. Cross bars represent homologous gene replacement event. (B) Genomic DNA PCRs of the WT, corresponding deletion and complementation strains with oligos F548/549. In each scenario, *biA* locus contains corresponding *fbx*::*gfp fusion*. M, marker; kb, kilobase. Arrows indicate approximate sizes of *fbx*::*gfp* fusions in biotin locus.
(TIF)

S5 Fig Developmental and growth phenotypes of *73* viable *fbx* gene deletions. Upper panels show 4 days grown *fbx* mutants as well as a WT strain under continuous white light. Lower panels show stereomicroscope close-up photos of *fbx* mutant developments. Except for *fbx25* (*sconB*), all *fbx* mutants are viable, 7 of which exhibit strong developmental phenotypes, including lack of light response, lack of ascospores and secondary metabolite changes (*fbx15*, *23*, *27*, *38*, *72* and *74*). Point inoculated fresh 5x10^3^ spores were inoculated on plates and grown under light for 4 days at 37°C.
(TIF)

S6 Fig Growth of WT, developmental *fbx* deletion and complementation strains. Growth of WT, f*bx15*Δ, *fbx17*Δ, *fbx23*Δ, *fbx27*Δ, *fbx38*Δ, *fbx72*Δ and *fbx74*Δ along with *fbx*::*gfp* fusion strains (comp.). Strains (5x10^3^ spores) were grown on GMM plates at 37°C for 4 days under continuous white light.
(TIF)

S7 Fig Growth and sporulation levels of *fbx* mutants on alternative carbon sources. Two-dimensional heat map displays the overall growth and sporulation patterns of the *fbx* mutants on different carbon sources, containing mono-, di-saccharides and alcohols. Left panel indicates radial growth, the right panel shows sporulation levels on different carbon sources (All carbon sources were used as 1% w/v). Colour coding is given at the bottom of the Figure. Developmentally influenced *fbx* deletion strains *fbx15*, *fbx27*, *fbx38*, *fbx72* and *74* manifest phenotypes in almost all carbon sources. Several *fbx* mutants display mild increase in sporulation (*fbx16*, *18*, *20*, *21*, *22*, *26*, *28*, *29*, *52*, *59 to 65*). Point inoculated 5x10^3^ fungal spores were incubated on plates containing different carbon sources at 37°C for 3 days under continuous light conditions. Radial growth and asexual sporulation was measured from three independent plates (P≤0.01).
(TIF)

S8 Fig Growth of WT, developmental *fbx* deletion and complementation strains under oxidative and osmotic stress. (A) Inhibition zones of WT, *fbx15*Δ, *fbx23*Δ, *fbx27*Δ, *fbx38*Δ, *fbx72*Δ and *fbx74*Δ along with *fbx*::*gfp* fusion strains (comp.) under oxidative stress, 10% hydrogen peroxide (upper panel) and 50 mM menadione conditions. Paper disks were impregnated with 20 μl 10% hydrogen peroxide or 50 mM menadione. (B) Growth of the WT, *fbx15*Δ, *fbx17*Δ, *fbx23*Δ, *fbx27*Δ, *fbx38*Δ, *fbx72*Δ and *fbx74*Δ along with *fbx*::*gfp* fusion strains under osmotic stress conditions sodium and potassium chloride (1000 mM) at 37°C for 4 days. (C) Quantification of inhibition zones around nocodazole discs and colony size on cell wall stress agent calcofluor white. Paper disks were impregnated with 20 μl 50 μg Nocodazole. *fbx10*Δ, *fbx27*Δ, *fbx42*Δ, *fbx44*Δ, *fbx58*Δ show increased resistance (smaller inhibition zone) to nocodazole. *Fbx2*Δ, *fbx15*Δ, *fbx18*Δ, *fbx27*Δ, *fbx38*Δ, *fbx51*Δ, *fbx59*Δ, *fbx69*Δ, *fbx72*Δ, *fbx74*Δ show increased sensitivity (lower colony diameter) to calcoflour white (20 μg / ml). For disc experiments, strains (1x10^6^ spores) were grown on GMM plates at 37°C for 2 days under continuous white light. For plate experiments 5x10^3^ spores were grown on GMM plates at 37°C for 4 days under continuous white light.
(TIF)

S9 Fig Cellular localizations of the 73 F-box proteins. Subcellular localizations of the F-box proteins were observed under vegetative growth conditions. DRAQ5 was used to stain nuclei red, FM4-64 plasma membrane and SecA-mRFP fusion endoplasmic reticulum in red. For localization of the Fbx-GFP fusions, 400–500 spores were grown in liquid media for 16–20 hours at 30°C. Lack of F-box domains in several F-box proteins did not influence the subcellular localizations of the F-box proteins. NUC: Nucleus, MER: Merged, PM: Plasma membrane, ER: Endoplasmic reticulum.
(TIF)

S10 Fig Elimination of F-box domain from important F-box proteins. Complete amino acid sequences of Fbx15, 23, 25 and 27 are given. Squares on amino acid sequences for each protein represent the putative F-box domains. Yellow shades indicate the deleted residues. Predicted F-box domains of Fbx15 (4–50 aa), Fbx23 (105–152 aa), Fbx25 (177–226 aa), Fbx27 (132–180 aa) were deleted.
(TIF)

S11 Fig Silver stained SDS polyacrylamide gels of GFP TRAP eluates from 49 F-box-GFP fusion proteins. Final eluates of F-box-GFP fusions after GFP TRAP purification were run on 10% SDS polyacrylamide gel. Fat band in several gels represent only GFP control. ΔFbx15, ΔFbx17, ΔFbx23, ΔFbx25, ΔFbx27, ΔFbx40 represent GFP TRAP purifications performed without the F-box protein domains in the respective proteins. Developmentally important F-box proteins Fbx15, 23, 25, 27 and 38 (Fbx72 and 74 not shown here) were treated with two stress conditions, osmotic (NaCl) and oxidative (H_2_O_2_), respectively. Black arrows indicate expected sizes of Fbx-GFP fusion proteins.
(TIF)

S12 Fig VeA-GFP localizes to nuclei independent of Fbx23. 2 000 spores were inoculated in liquid minimal medium containing microscopic chambers (Ibidi) and incubated for 18 h in light at 37°C. Microscopic pictures were taken with the Plan-Apochromat 100x/1.4 oil objective. DAPI was used to visualize nuclei. Size bars indicate 10 μm.
(TIF)

S1 Table SkpA interacting proteins during 24h vegetative growth. (XLSX)

S2 Table SkpA interacting proteins during 24h asexual development in the light. (XLSX)

S3 Table SkpA interacting proteins during 24h sexual development in the dark. (XLSX)

S4 Table SkpA interacting proteins during 48h sexual development in the dark. (XLSX)

S5 Table SkpA interacting proteins under osmotic stress (0.5 M NaCl). (XLSX)

S6 Table SkpA interacting proteins under oxidative stress (5mM H2O2). (XLSX)

S7 Table SkpA interacting proteins under oxidative stress (80mm Menadione). (XLSX)

S8 Table Free GFP control (only GFP interacting proteins). (XLSX)

S9 Table SkpA and CulA interacting proteins during vegetative growth. (XLSX)

S10 Table F-box domain orthologs in major eukaryotic organisms. (XLSX)

S11 Table List of F-box interacting proteins. (XLSX)

S12 Table F-box associated proteins based on their appearance in different F-box purifications. (XLSX)

S13 Table Grouping of F-box associated proteins based on their cellular localization and functions. (XLSX)

S14 Table Regulatory proteins associated with F-box proteins. (XLSX)

S15 Table Strains used in this study. (XLSX)

S16 Table Deletion cassettes. (XLSX)

S17 Table Oligos. (XLSX)

S18 Table GFP fusion plasmids. (XLSX)

S19 Table Plasmids. (XLSX)

S1 Text Supplementary materials and methods. (DOCX)

S1 Data LCMS method information. (XLSX)

## References

[1] Denning DW, Pleuvry A, Cole DC. Global burden of allergic bronchopulmonary aspergillosis with asthma and its complication chronic pulmonary aspergillosis in adults. Med Mycol. 2013;51: 361–370. doi: 10.3109/13693786.2012.738312 23210682

[2] Galagan JE, Calvo SE, Cuomo C, Ma L, Wortman JR, Batzoglou S, et al. Sequencing of Aspergillus nidulans and comparative analysis with A. fumigatus and A. oryzae. Nature. 2005;438: 1105–1115. doi: 10.1038/nature04341 16372000

[3] Bayram Ö, Braus GH. Coordination of secondarymetabolism and development in fungi: the velvet familyof regulatory proteins. FEMS Microbiol Rev. 2012;36: 1–24. doi: 10.1111/j.1574-6976.2011.00285.x 21658084

[4] Riquelme M, Aguirre J, Bartnicki–García S, Braus GH, Feldbrügge M, Fleig U, et al. Fungal morphogenesis, from the polarized growth of hyphae to complex reproduction and infection structures. Microbiol Mol Biol Rev. 2018;82: e00068–17. doi: 10.1128/MMBR.00068-17 29643171 PMC5968459

[5] Rodriguez–Romero J, Hedtke M, Kastner C, Müller S, Fischer R. Fungi, hidden in soil or up in the air: light makes a difference. Annu Rev Microbiol. 2010;64: 585–610. doi: 10.1146/annurev.micro.112408.134000 20533875

[6] Keller NP, Turner G, Bennett JW. Fungal secondary metabolism–from biochemistry to genomics. 2005. doi: 10.1038/nrmicro1286 16322742

[7] Brakhage AA. Regulation of fungal secondary metabolism. Nat Rev Microbiol. 2013;11: 21–32. doi: 10.1038/nrmicro2916 23178386

[8] Bayram Ö, Krappmann S, Ni M, Bok JW, Helmstaedt K, Valerius O, et al. VelB/VeA/LaeA complex coordinates light signal with fungal development and secondary metabolism. Science. 2008;320: 1504–6. doi: 10.1126/science.1155888 18556559

[9] Komander D, Rape M. The ubiquitin code. Annu Rev Biochem. 2012;81: 203–229. doi: 10.1146/annurev-biochem-060310-170328 22524316

[10] Gerke J, Köhler AM, Meister C, Thieme KG, Amoedo H, Braus GH. Coordination of fungal secondary metabolism and development. vol 2. In: Benz JP, Schipper K, editors. Genetics and Biotechnology The Mycota (A Comprehensive Treatise on Fungi as Experimental Systems for Basic and Applied Research). vol 2. Cham: vol 2. Springer; 2020. pp. 173–205. doi: 10.1007/978–3–030–49924–2_8

[11] von Zeska Kress MR, Harting R, Bayram Ö, Christmann M, Irmer H, Valerius O, et al. The COP9 signalosome counteracts the accumulation of cullin SCF ubiquitin E3 RING ligases during fungal development. Mol Microbiol. 2012;83: 1162–1177. doi: 10.1111/j.1365-2958.2012.07999.x 22329854

[12] Kolog Gulko M, Heinrich G, Gross C, Popova B, Valerius O, Neumann P, et al. Sem1 links proteasome stability and specificity to multicellular development. Brakhage AA, editor. PLOS Genet. 2018;14: e1007141. doi: 10.1371/journal.pgen.1007141 29401458 PMC5821377

[13] Peng J, Schwartz D, Elias JE, Thoreen CC, Cheng D, Marsischky G, et al. A proteomics approach to understanding protein ubiquitination. Nat Biotechnol. 2003;21: 921–926. doi: 10.1038/nbt849 12872131

[14] Chu X–L, Feng M–G, Ying S–H. Qualitative ubiquitome unveils the potential significances of protein lysine ubiquitination in hyphal growth of Aspergillus nidulans. Curr Genet. 2016;62: 191–201. doi: 10.1007/s00294-015-0517-7 26328806

[15] Skaar JR, Pagan JK, Pagano M. Mechanisms and function of substrate recruitment by F–box proteins. Nat Rev Mol Cell Biol. 2013;14: 369–81. doi: 10.1038/nrm3582 23657496 PMC3827686

[16] Hua Z, Vierstra RD. The Cullin–RING ubiquitin–protein ligases. Annu Rev Plant Biol. 2011;62: 299–334. doi: 10.1146/annurev-arplant-042809-112256 21370976

[17] Craig KL, Tyers M. The F–box: A new motif for ubiquitin dependent proteolysis in cell cycle regulation and signal transduction. Prog Biophys Mol Biol. 1999;72: 299–328. doi: 10.1016/s0079-6107(99)00010-3 10581972

[18] Schmidt MW, McQuary PR, Wee S, Hofmann K, Wolf DA. F–Box–directed CRL complex assembly and regulation by the CSN and CAND1. Mol Cell. 2009;35: 586–597. doi: 10.1016/j.molcel.2009.07.024 19748355 PMC2779159

[19] Wu S, Zhu W, Nhan T, Toth JI, Petroski MD, Wolf DA. CAND1 controls in vivo dynamics of the cullin 1–RING ubiquitin ligase repertoire. Nat Commun. 2013;4: 1642. doi: 10.1038/ncomms2636 23535663 PMC3637025

[20] Lingaraju GM, Bunker RD, Cavadini S, Hess D, Hassiepen U, Renatus M, et al. Crystal structure of the human COP9 signalosome. Nature. 2014;512: 161–165. doi: 10.1038/nature13566 25043011

[21] Wei N, Chamovitz DA, Deng XW. Arabidopsis COP9 is a component of a novel signaling complex mediating light control of development. Cell. 1994;78: 117–24. doi: 10.1016/0092-8674(94)90578-9 8033203

[22] Helmstaedt K, Schwier EU, Christmann M, Nahlik K, Westermann M, Harting R, et al. Recruitment of the inhibitor Cand1 to the cullin substrate adaptor site mediates interaction to the neddylation site. Mol Biol Cell. 2011;22: 153–164. doi: 10.1091/mbc.E10-08-0732 21119001 PMC3016973

[23] Tomoda K, Yoneda–Kato N, Fukumoto A, Yamanaka S, Kato J. Multiple functions of Jab1 are required for early embryonic development and growth potential in mice. J Biol Chem. 2004;279: 43013–43018. doi: 10.1074/jbc.M406559200 15299027

[24] Busch S, Eckert SE, Krappmann S, Braus GH. The COP9 signalosome is an essential regulator of development in the filamentous fungus *Aspergillus nidulans*. Mol Microbiol. 2003;49: 717–730. doi: 10.1046/j.1365–2958.2003.03612.x 12864854

[25] Busch S, Schwier EU, Nahlik K, Bayram Ö, Helmstaedt K, Draht OW, et al. An eight–subunit COP9 signalosome with an intact JAMM motif is required for fungal fruit body formation. Proc Natl Acad Sci U S A. 2007;104: 8089–94. doi: 10.1073/pnas.0702108104 17470786 PMC1876576

[26] Beckmann EA, Köhler AM, Meister C, Christmann M, Draht OW, Rakebrandt N, et al. Integration of the catalytic subunit activates deneddylase activity *in vivo* as final step in fungal COP9 signalosome assembly. Mol Microbiol. 2015;97: 110–124. doi: 10.1111/mmi.13017 25846252

[27] Nahlik K, Dumkow M, Bayram Ö, Helmstaedt K, Busch S, Valerius O, et al. The COP9 signalosome mediates transcriptional and metabolic response to hormones, oxidative stress protection and cell wall rearrangement during fungal development. Mol Microbiol. 2010;78: 964–979. doi: 10.1111/j.1365-2958.2010.07384.x 21062371

[28] Köhler AM, Harting R, Langeneckert AE, Valerius O, Gerke J, Meister C, et al. Integration of fungus–specific CandA–C1 into a trimeric CandA complex allowed splitting of the gene for the conserved receptor exchange factor of cullinA E3 ubiquitin ligases in Aspergilli. Di Pietro A, editor. MBio. 2019;10. doi: 10.1128/mBio.01094-19 31213557 PMC6581859

[29] Christmann M, Schmaler T, Gordon C, Huang X, Bayram O, Schinke J, et al. Control of multicellular development by the physically interacting deneddylases DEN1/DenA and COP9 signalosome. Heitman J, editor. PLoS Genet. 2013;9: e1003275. doi: 10.1371/journal.pgen.1003275 23408908 PMC3567183

[30] Jonkers W, Rep M. Lessons from Fungal F–Box Proteins. Eukaryot Cell. 2009;8: 677–695. doi: 10.1128/EC.00386-08 19286981 PMC2681605

[31] Goh P–Y, Surana U. Cdc4, a Protein Required for the Onset of S Phase, Serves an Essential Function during G 2 /M Transition in Saccharomyces cerevisiae. Mol Cell Biol. 1999;19: 5512–5522. doi: 10.1128/MCB.19.8.5512 10409741 PMC84393

[32] Draht OW, Busch S, Hofmann K, Braus–Stromeyer S, Helmstaedt K, Goldman GH, et al. Amino Acid Supply of Aspergillus. In: Osmani SA, Goldman GH, editors. The Aspergilli: Genomics, Medical Aspects, Biotechnology, and Research Methods. CRC Press Taylor & Francis, USA; 2007. pp. 143–175.

[33] Skaar JR, D’Angiolella V, Pagan JK, Pagano M. SnapShot: F Box proteins II. Cell. 2009;137: 1358.e1–1358.e2. doi: 10.1016/j.cell.2009.05.040 19563764

[34] Hua Z, Zou C, Shiu S–H, Vierstra RD. Phylogenetic comparison of F–Box (FBX) gene superfamily within the plant kingdom reveals divergent evolutionary histories indicative of genomic drift. Umen J, editor. PLoS One. 2011;6: e16219. doi: 10.1371/journal.pone.0016219 21297981 PMC3030570

[35] Stefanowicz K, Lannoo N, Van Damme EJM. Plant F–box proteins–judges between life and death. Critical Reviews in Plant Sciences Taylor & Francis; Nov 2, 2015 pp. 523–552. doi: 10.1080/07352689.2015.1024566

[36] Zheng N, Wang Z, Wei W. Ubiquitination–mediated degradation of cell cycle–related proteins by F–box proteins. Int J Biochem Cell Biol. 2016;73: 99–110. doi: 10.1016/j.biocel.2016.02.005 26860958 PMC4798898

[37] Wu T, Fan C–L, Han L–T, Guo Y–B, Liu T–B. Role of F–box Protein Cdc4 in Fungal Virulence and Sexual Reproduction of Cryptococcus neoformans. Front Cell Infect Microbiol. 2022;11. doi: 10.3389/fcimb.2021.806465 35087766 PMC8787122

[38] Masso–Silva J, Espinosa V, Liu T–B, Wang Y, Xue C, Rivera A. The F–Box protein Fbp1 shapes the immunogenic potential of *Cryptococcus neoformans*. Dromer F, editor. MBio. 2018;9. doi: 10.1128/mBio.01828–17 PMC5760740 29317510

[39] Liu T–B, Xue C. The Ubiquitin–Proteasome System and F–box Proteins in Pathogenic Fungi. Mycobiology. 2011;39: 243–248. doi: 10.5941/MYCO.2011.39.4.243 22783111 PMC3385136

[40] He Q, Cheng P, Yang Y, He Q, Yu H, Liu Y. FWD1–mediated degradation of FREQUENCY in Neurospora establishes a conserved mechanism for circadian clock regulation. EMBO J. 2003;22: 4421–30. doi: 10.1093/emboj/cdg425 12941694 PMC202367

[41] de Assis LJ, Ulas M, Ries LNA, El Ramli NAM, Sarikaya–Bayram O, Braus GH, et al. Regulation of *Aspergillus nidulans* CreA–mediated catabolite repression by the F–Box proteins Fbx23 and Fbx47. MBio. 2018;9: 1–19. doi: 10.1128/mBio.00840–18 PMC6016232 29921666

[42] Gil–Sánchez M del M, Cea–Sánchez S, Luque EM, Cánovas D, Corrochano LM. Light regulates the degradation of the regulatory protein VE–1 in the fungus Neurospora crassa. BMC Biol. 2022;20: 149. doi: 10.1186/s12915-022-01351-x 35761233 PMC9238092

[43] Jöhnk B, Bayram Ö, Abelmann A, Heinekamp T, Mattern DJ, Brakhage AA, et al. SCF ubiquitin ligase F–box protein Fbx15 controls nuclear co–repressor localization, stress response and virulence of the human pathogen *Aspergillus fumigatus*. PLoS Pathog. 2016;12: e1005899. doi: 10.1371/journal.ppat.1005899 27649508 PMC5029927

[44] Krappmann S, Jung N, Medic B, Busch S, Prade RA, Braus GH. The *Aspergillus nidulans* F–box protein GrrA links SCF activity to meiosis. Mol Microbiol. 2006;61: 76–88. doi: 10.1111/j.1365–2958.2006.05215.x 16824096

[45] Karahoda B, Pardeshi L, Ulas M, Dong Z, Shirgaonkar N, Guo S, et al. The KdmB–EcoA–RpdA–SntB chromatin complex binds regulatory genes and coordinates fungal development with mycotoxin synthesis. Nucleic Acids Res. 2022;50: 9797–9813. doi: 10.1093/nar/gkac744 36095118 PMC9508808

[46] Sarikaya–Bayram Ö, Bayram Ö, Feussner K, Kim J–H, Kim H–S, Kaever A, et al. Membrane–Bound Methyltransferase Complex VapA–VipC–VapB Guides Epigenetic Control of Fungal Development. Dev Cell. 2014;29: 406–420. doi: 10.1016/j.devcel.2014.03.020 24871947

[47] Hermand D. F–box proteins: More than baits for the SCF? Cell Division. BioMed Central; 2006. p. 30. doi: 10.1186/1747–1028–1–30 PMC1712225 17166256

[48] Chowdhury M, Enenkel C. Intracellular dynamics of the ubiquitin–proteasome–system. F1000Research. 2015;4: 367. doi: 10.12688/f1000research.6835.2 26339477 PMC4544378

[49] De Souza CP, Hashmi SB, Osmani AH, Andrews P, Ringelberg CS, Dunlap JC, et al. Functional Analysis of the Aspergillus nidulans Kinome. Yu J–H, editor. PLoS One. 2013;8: e58008. doi: 10.1371/journal.pone.0058008 23505451 PMC3591445

[50] Richie DL, Miley MD, Bhabhra R, Robson GD, Rhodes JC, Askew DS. The Aspergillus fumigatus metacaspases CasA and CasB facilitate growth under conditions of endoplasmic reticulum stress. Mol Microbiol. 2006;63: 591–604. doi: 10.1111/j.1365-2958.2006.05534.x 17176258

[51] Meister C, Thieme KG, Thieme S, Köhler AM, Schmitt K, Valerius O, et al. COP9 Signalosome Interaction with UspA/Usp15 Deubiquitinase Controls VeA–Mediated Fungal Multicellular Development. Biomolecules. 2019;9: 238. doi: 10.3390/biom9060238 31216760 PMC6627422

[52] Ye Y, Rape M. Building ubiquitin chains: E2 enzymes at work. Nat Rev Mol Cell Biol. 2009;10: 755–764. doi: 10.1038/nrm2780 19851334 PMC3107738

[53] Gerke J, Köhler AM, Wennrich J–P, Große V, Shao L, Heinrich AK, et al. Biosynthesis of Antibacterial Iron–Chelating Tropolones in Aspergillus nidulans as Response to Glycopeptide–Producing Streptomycetes. Front Fungal Biol. 2022;2: 68. doi: 10.3389/ffunb.2021.777474 PMC10512232 37744088

[54] Liu L, Sasse C, Dirnberger B, Valerius O, Fekete–Szücs E, Harting R, et al. Secondary metabolites of Hülle cells mediate protection of fungal reproductive and overwintering structures against fungivorous animals. Elife. 2021;10. doi: 10.7554/eLife.68058 34635205 PMC8510581

[55] Kuroda H, Yanagawa Y, Takahashi N, Horii Y, Matsui M. A comprehensive analysis of interaction and localization of Arabidopsis SKP1–LIKE (ASK) and F–Box (FBX) proteins. Rahman A, editor. PLoS One. 2012;7: e50009. doi: 10.1371/journal.pone.0050009 23166809 PMC3499479

[56] Lu Z, Hunter T. Degradation of activated protein kinases by ubiquitination. Annu Rev Biochem. 2009;78: 435–75. doi: 10.1146/annurev.biochem.013008.092711 19489726 PMC2776765

[57] Zhao Y, Xiong X, Sun Y. DEPTOR, an mTOR inhibitor, is a physiological substrate of SCFβTrCP E3 ubiquitin ligase and regulates survival and autophagy. Mol Cell. 2011;44: 304–316. doi: 10.1016/j.molcel.2011.08.029 22017876 PMC3216641

[58] Seol JH, Shevchenko A, Shevchenko A, Deshaies RJ. Skp1 forms multiple protein complexes, including RAVE, a regulator of V–ATPase assembly. Nat Cell Biol. 2001;3: 384–391. doi: 10.1038/35070067 11283612

[59] Lehmann A, Katayama S, Harrison C, Dhut S, Kitamura K, McDonald N, et al. Molecular interactions of fission yeast Skp1 and its role in the DNA damage checkpoint. Genes to Cells. 2004;9: 367–382. doi: 10.1111/j.1356-9597.2004.00730.x 15147268

[60] Dezfulian MH, Soulliere DM, Dhaliwal RK, Sareen M, Crosby WL. The SKP1–Like gene family of Arabidopsis exhibits a high degree of differential gene expression and gene product interaction during development. Bassham D, editor. PLoS One. 2012;7: e50984. doi: 10.1371/journal.pone.0050984 23226441 PMC3511428

[61] Patton EE, Willems AR, Tyers M. Combinatorial control in ubiquitin–dependent proteolysis: don’t Skp the F–box hypothesis. Trends Genet. 1998;14: 236–43. doi: 10.1016/s0168-9525(98)01473-5 9635407

[62] Mathur R, Yen JL, Kaiser P. Skp1 independent function of Cdc53/Cul1 in F–box protein homeostasis. Toczyski DP, editor. PLOS Genet. 2015;11: e1005727. doi: 10.1371/journal.pgen.1005727 26656496 PMC4675558

[63] Pradhan A, Ma Q, de Assis LJ, Leaves I, Larcombe DE, Rodriguez Rondon A V., et al. Anticipatory Stress Responses and Immune Evasion in Fungal Pathogens. Trends Microbiol. 2021;29: 416–427. doi: 10.1016/j.tim.2020.09.010 33059975

[64] Bayram Ö, Bayram ÖS, Ahmed YL, Maruyama J, Valerius O, Rizzoli SO, et al. The *Aspergillus nidulans* MAPK module AnSte11–Ste50–Ste7–Fus3 controls development and secondary metabolism. Madhani HD, editor. PLoS Genet. 2012;8: e1002816. doi: 10.1371/journal.pgen.1002816 22829779 PMC3400554

