# Supplementary figures and images for: F-box receptor mediated control of substrate stability and subcellular location organizes cellular development of Aspergillus nidulans
Source: PLoS Genet. 2022 Dec 12;18(12):e1010502. doi: 10.1371/journal.pgen.1010502 (PMC9744329; doi:10.1371/journal.pgen.1010502)

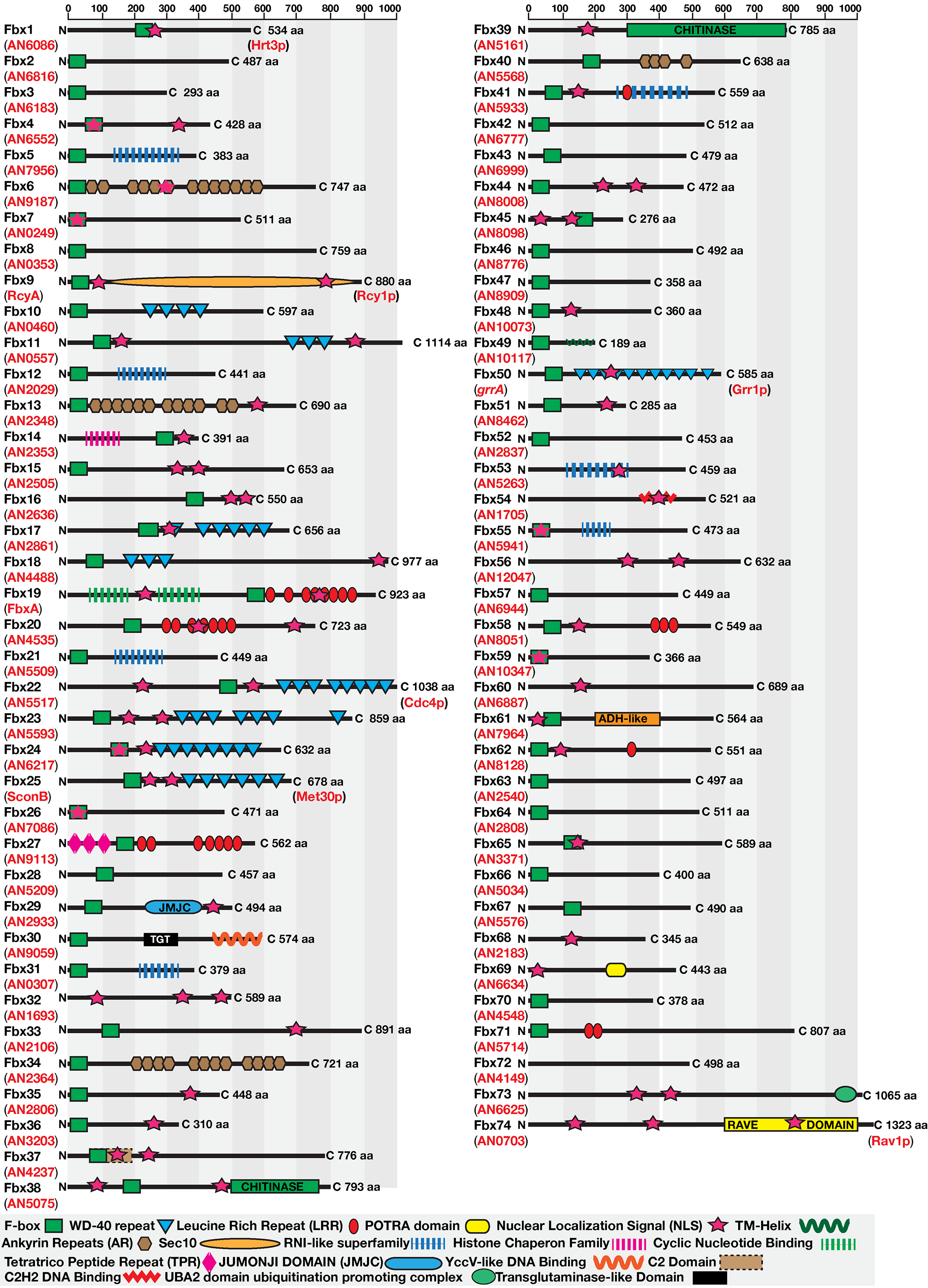

Supplement: S1 Fig — Top scale indicates the sizes of the proteins in amino acids (from 0 to 1000). Proteins exceeding the maximum length scale are Fbx11, 22, 73 and 74. Locus identities are given on the left-hand side as AN-numbers. Yeast homologs are given on the right-hand side. Identified F-box domains are indicated as green squares, common motifs such as Leucine Rich Repeats (LRR) or WD-40 domains found in F-box proteins are shown as blue triangles and red oval spheres, respectively. Red star represents Nuclear Localization Signal (NLS). Brown hexagons represent Ankyrin Repeats (AR). Polypeptide-transport-associated (POTRA), Transmembrane Helix (TM-Helix), Regulator of the ATPase of vacuolar and endosomal membranes (RAVE). Rest of the descriptions of the further identified domains present on F-box proteins are given at the bottom of the Figure. (TIF) [file pgen.1010502.s001.tif]

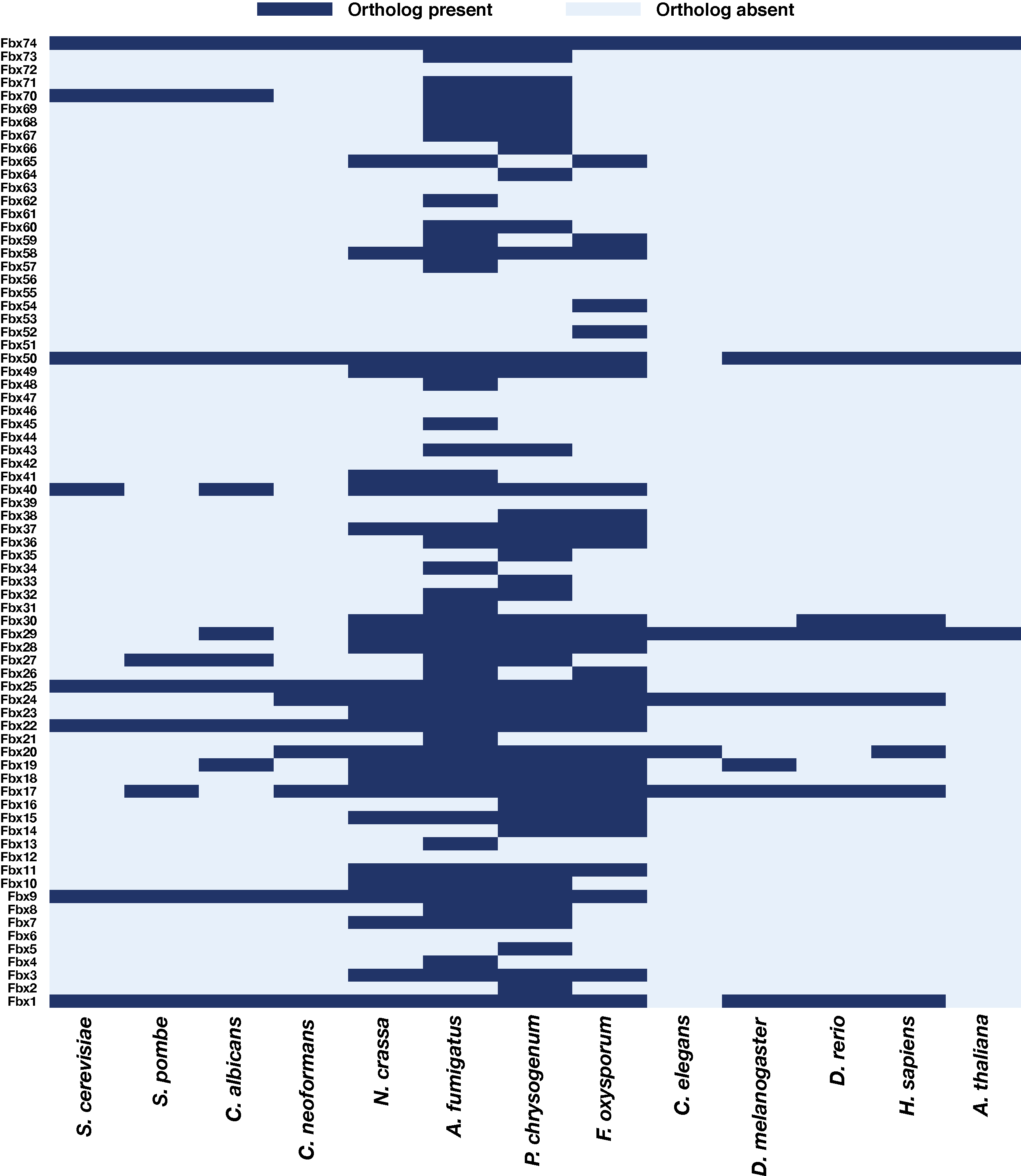

Supplement: S2 Fig — Orthologs of 74 F-box domain proteins were determined from single-celled yeasts, filamentous fungi to human using a reciprocal best BLAST hits strategy. A. nidulans F-box proteins have more common orthologs in filamentous fungi, and less in single-celled yeast and human. Accession numbers or locus ID were given in S10 Table. (TIF) [file pgen.1010502.s002.tif]

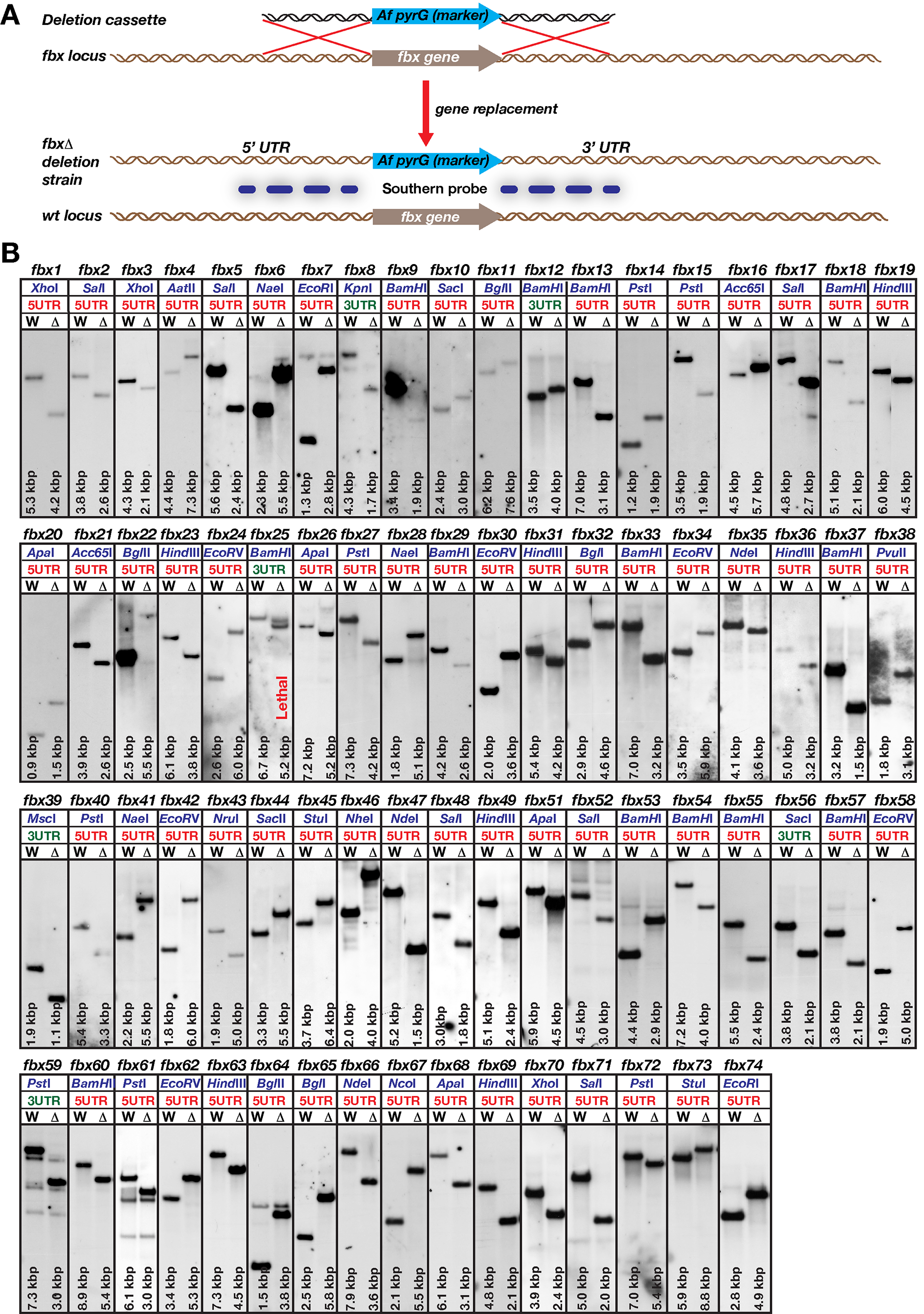

Supplement: S3 Fig — (A) An illustrative depiction of the deletion events via homologous gene replacement. 5´ UTR: five prime untranslated region, 3´ UTR: three prime untranslated region. Dashed lines with shadows represent either 5´ UTR or 3´ UTR hybridizing Southern probes (ranging from 500 to 800 bps). Aspergillus fumigatus-derived pyrG gene, AfpyrG served as a selection marker for the deletion events. (B) Southern hybridization results of 73 fbx gene deletion events. The fbx genes were given at the top of the blots, below of which restriction enzymes used for controlling each deletion event and Southern probes (5’ or 3’ UTR) were highlighted. Only fbx25 (encoding SconB) was lethal (double band one ectopic deletion cassette and one endogenous fbx25 locus). Sizes of the bands are given at the bottom of the blots in kbps. W: WT locus, Δ: respective fbx deletion. (TIF) [file pgen.1010502.s003.tif]

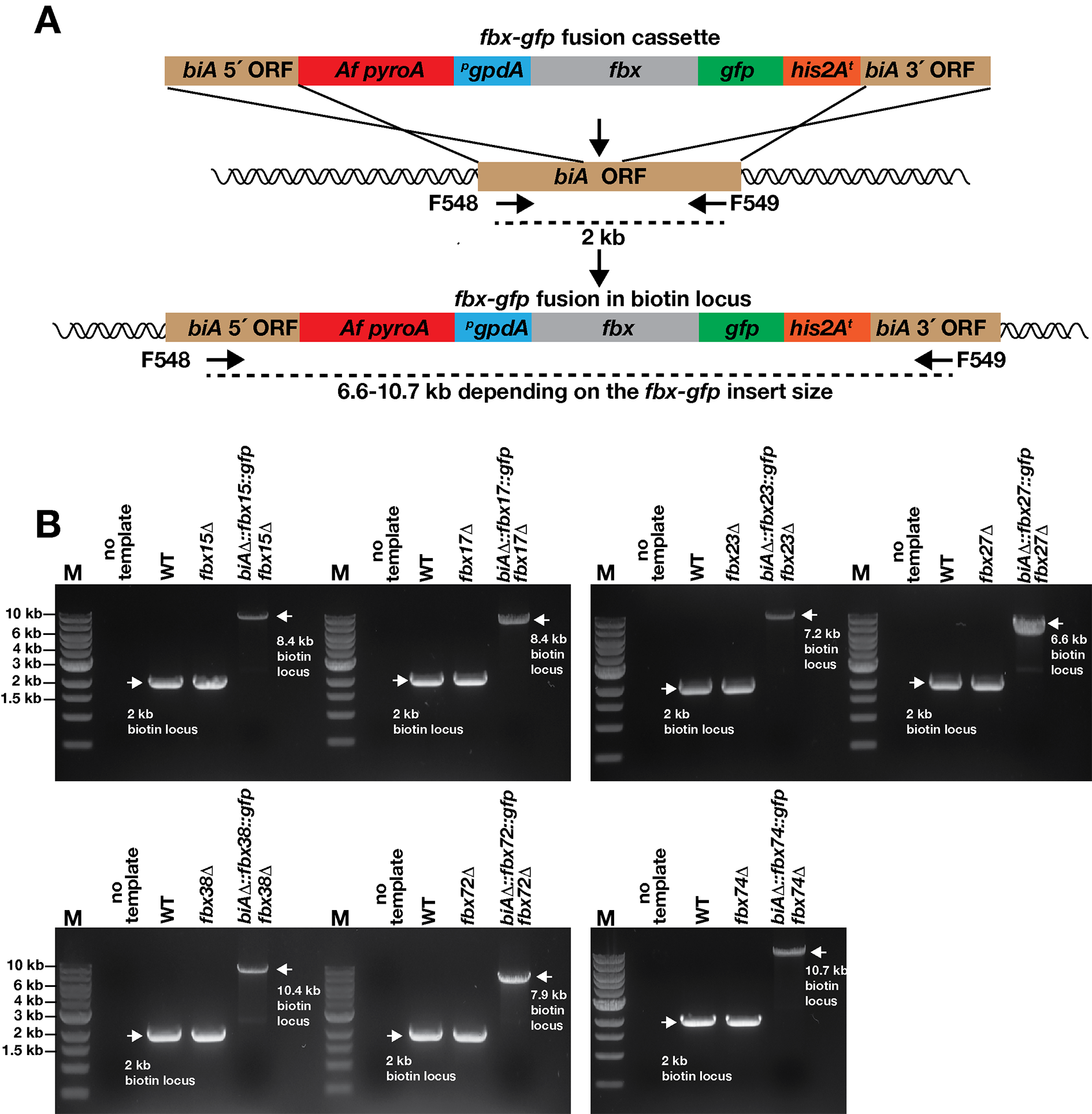

Supplement: S4 Fig — (A) Schematic representation of fbx::gfp cassettes, original biotin locus (biA) in WT and fbxΔ strains, and biotin locus in complementation strains. Cross bars represent homologous gene replacement event. (B) Genomic DNA PCRs of the WT, corresponding deletion and complementation strains with oligos F548/549. In each scenario, biA locus contains corresponding fbx::gfp fusion. M, marker; kb, kilobase. Arrows indicate approximate sizes of fbx::gfp fusions in biotin locus. (TIF) [file pgen.1010502.s004.tif]

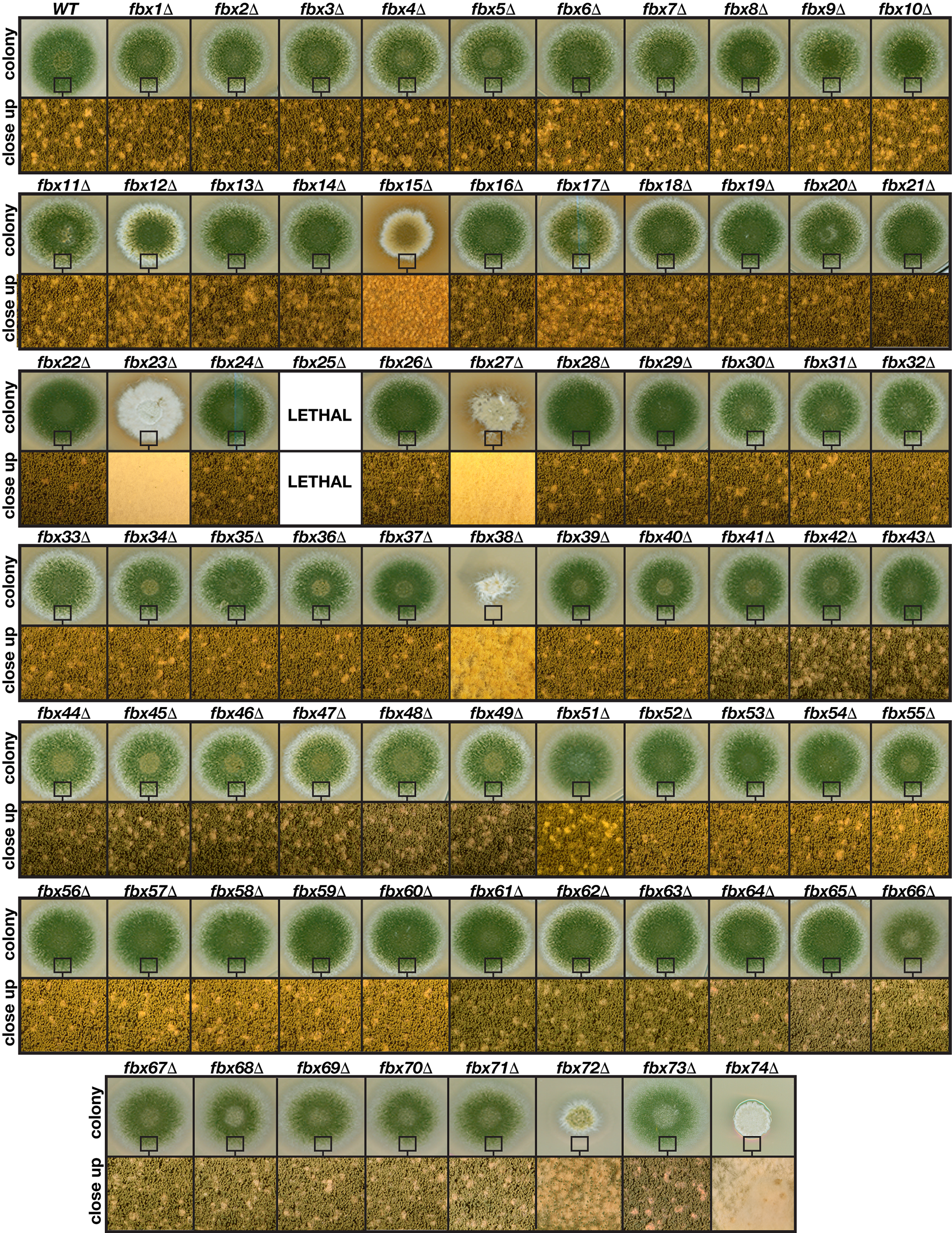

Supplement: S5 Fig — Upper panels show 4 days grown fbx mutants as well as a WT strain under continuous white light. Lower panels show stereomicroscope close-up photos of fbx mutant developments. Except for fbx25 (sconB), all fbx mutants are viable, 7 of which exhibit strong developmental phenotypes, including lack of light response, lack of ascospores and secondary metabolite changes (fbx15, 23, 27, 38, 72 and 74). Point inoculated fresh 5x10^3^ spores were inoculated on plates and grown under light for 4 days at 37°C. (TIF) [file pgen.1010502.s005.tif]

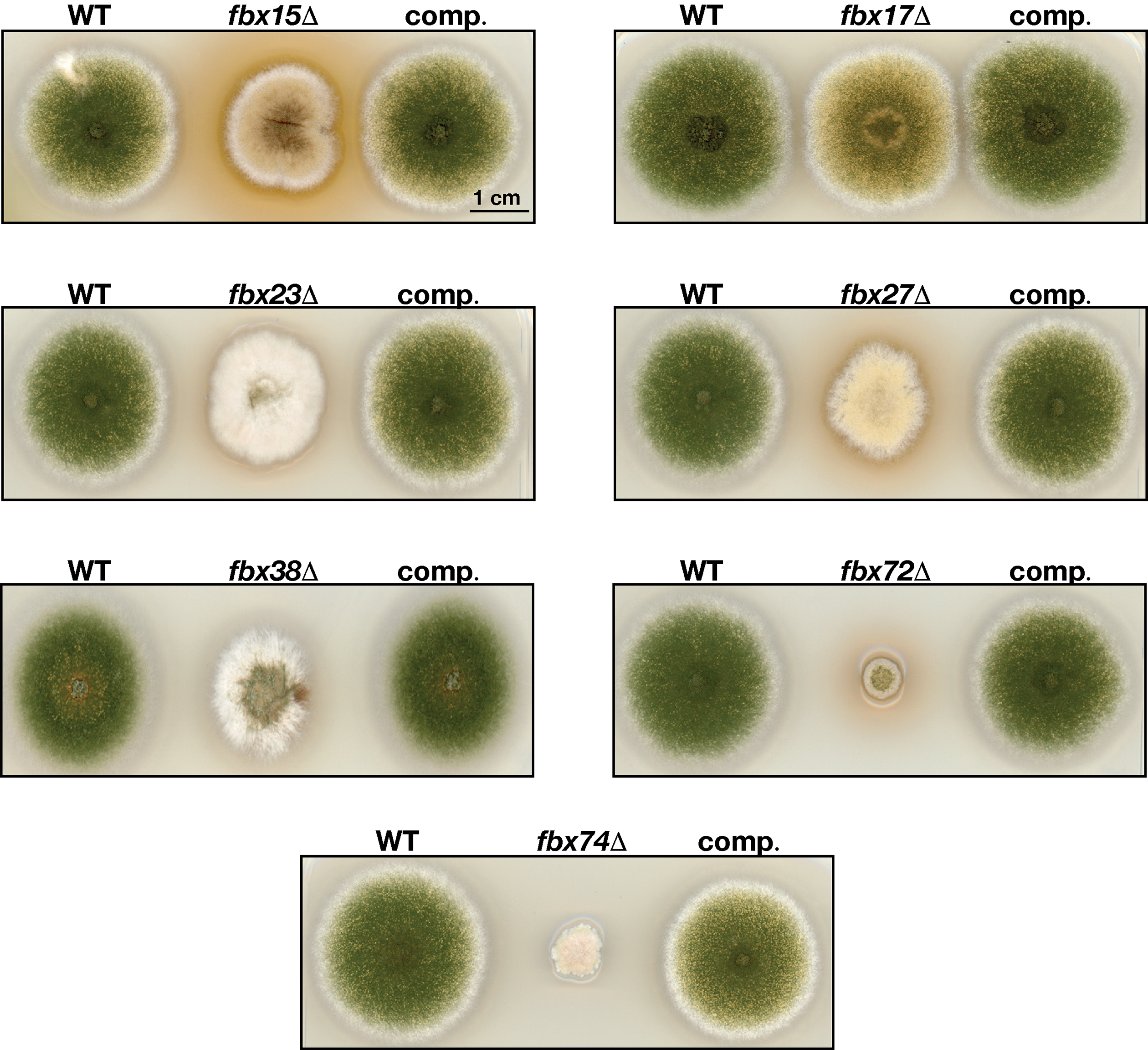

Supplement: S6 Fig — Growth of WT, fbx15Δ, fbx17Δ, fbx23Δ, fbx27Δ, fbx38Δ, fbx72Δ and fbx74Δ along with fbx::gfp fusion strains (comp.). Strains (5x10^3^ spores) were grown on GMM plates at 37°C for 4 days under continuous white light. (TIF) [file pgen.1010502.s006.tif]

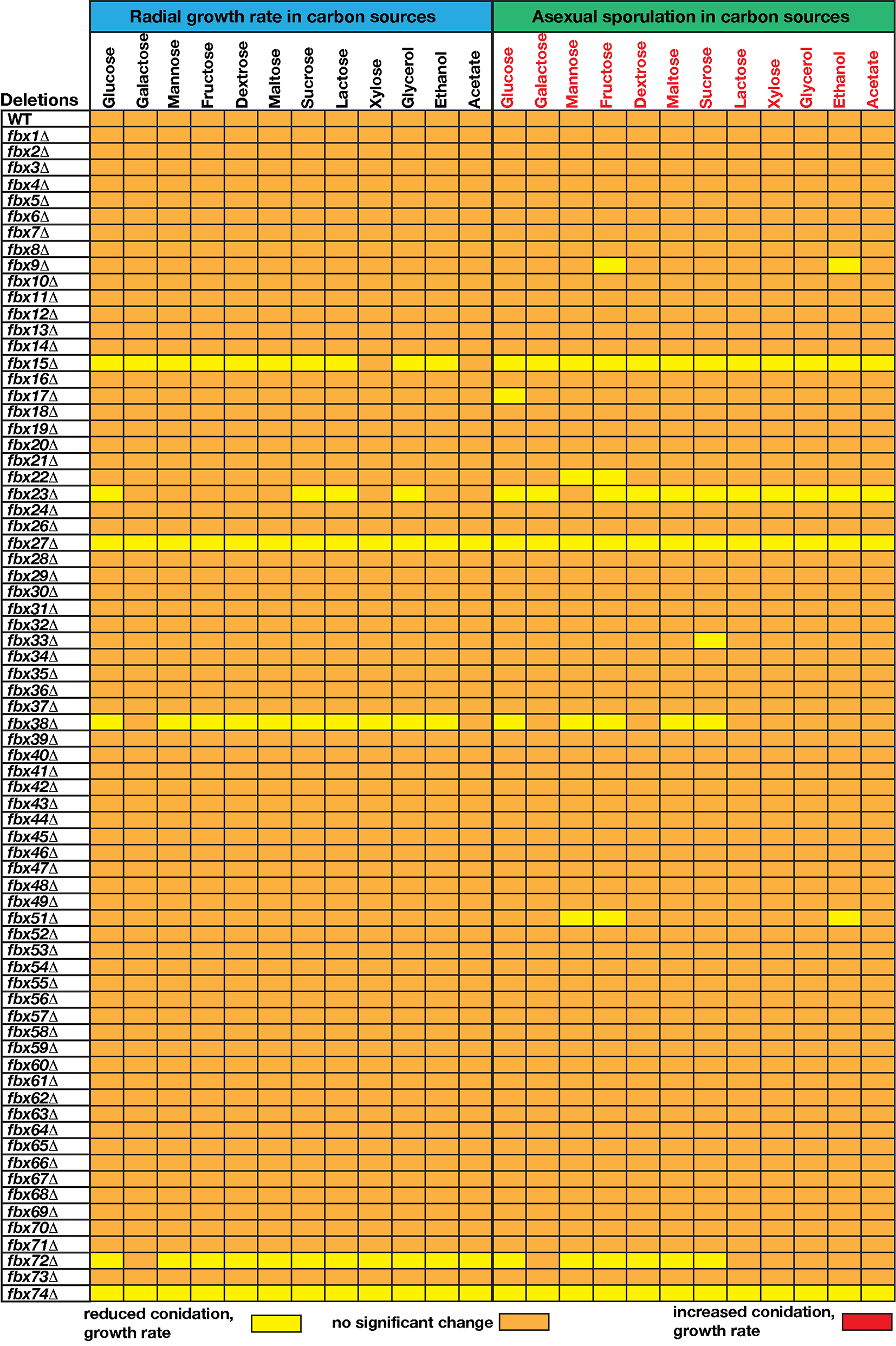

Supplement: S7 Fig — Two-dimensional heat map displays the overall growth and sporulation patterns of the fbx mutants on different carbon sources, containing mono-, di-saccharides and alcohols. Left panel indicates radial growth, the right panel shows sporulation levels on different carbon sources (All carbon sources were used as 1% w/v). Colour coding is given at the bottom of the Figure. Developmentally influenced fbx deletion strains fbx15, fbx27, fbx38, fbx72 and 74 manifest phenotypes in almost all carbon sources. Several fbx mutants display mild increase in sporulation (fbx16, 18, 20, 21, 22, 26, 28, 29, 52, 59 to 65). Point inoculated 5x10^3^ fungal spores were incubated on plates containing different carbon sources at 37°C for 3 days under continuous light conditions. Radial growth and asexual sporulation was measured from three independent plates (P≤0.01). (TIF) [file pgen.1010502.s007.tif]

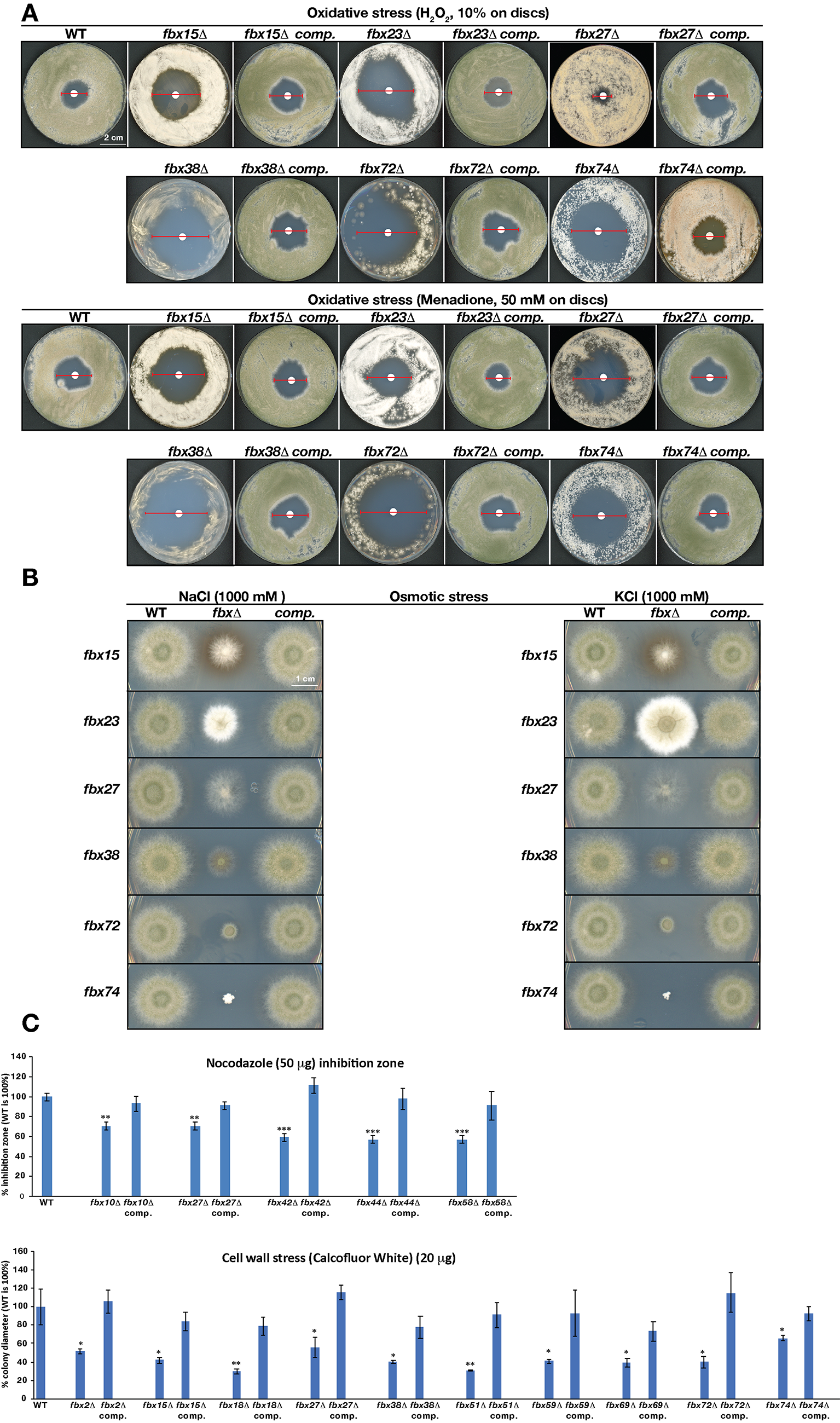

Supplement: S8 Fig — (A) Inhibition zones of WT, fbx15Δ, fbx23Δ, fbx27Δ, fbx38Δ, fbx72Δ and fbx74Δ along with fbx::gfp fusion strains (comp.) under oxidative stress, 10% hydrogen peroxide (upper panel) and 50 mM menadione conditions. Paper disks were impregnated with 20 μl 10% hydrogen peroxide or 50 mM menadione. (B) Growth of the WT, fbx15Δ, fbx17Δ, fbx23Δ, fbx27Δ, fbx38Δ, fbx72Δ and fbx74Δ along with fbx::gfp fusion strains under osmotic stress conditions sodium and potassium chloride (1000 mM) at 37°C for 4 days. (C) Quantification of inhibition zones around nocodazole discs and colony size on cell wall stress agent calcofluor white. Paper disks were impregnated with 20 μl 50 μg Nocodazole. fbx10Δ, fbx27Δ, fbx42Δ, fbx44Δ, fbx58Δ show increased resistance (smaller inhibition zone) to nocodazole. Fbx2Δ, fbx15Δ, fbx18Δ, fbx27Δ, fbx38Δ, fbx51Δ, fbx59Δ, fbx69Δ, fbx72Δ, fbx74Δ show increased sensitivity (lower colony diameter) to calcoflour white (20 μg / ml). For disc experiments, strains (1x10^6^ spores) were grown on GMM plates at 37°C for 2 days under continuous white light. For plate experiments 5x10^3^ spores were grown on GMM plates at 37°C for 4 days under continuous white light. (TIF) [file pgen.1010502.s008.tif]

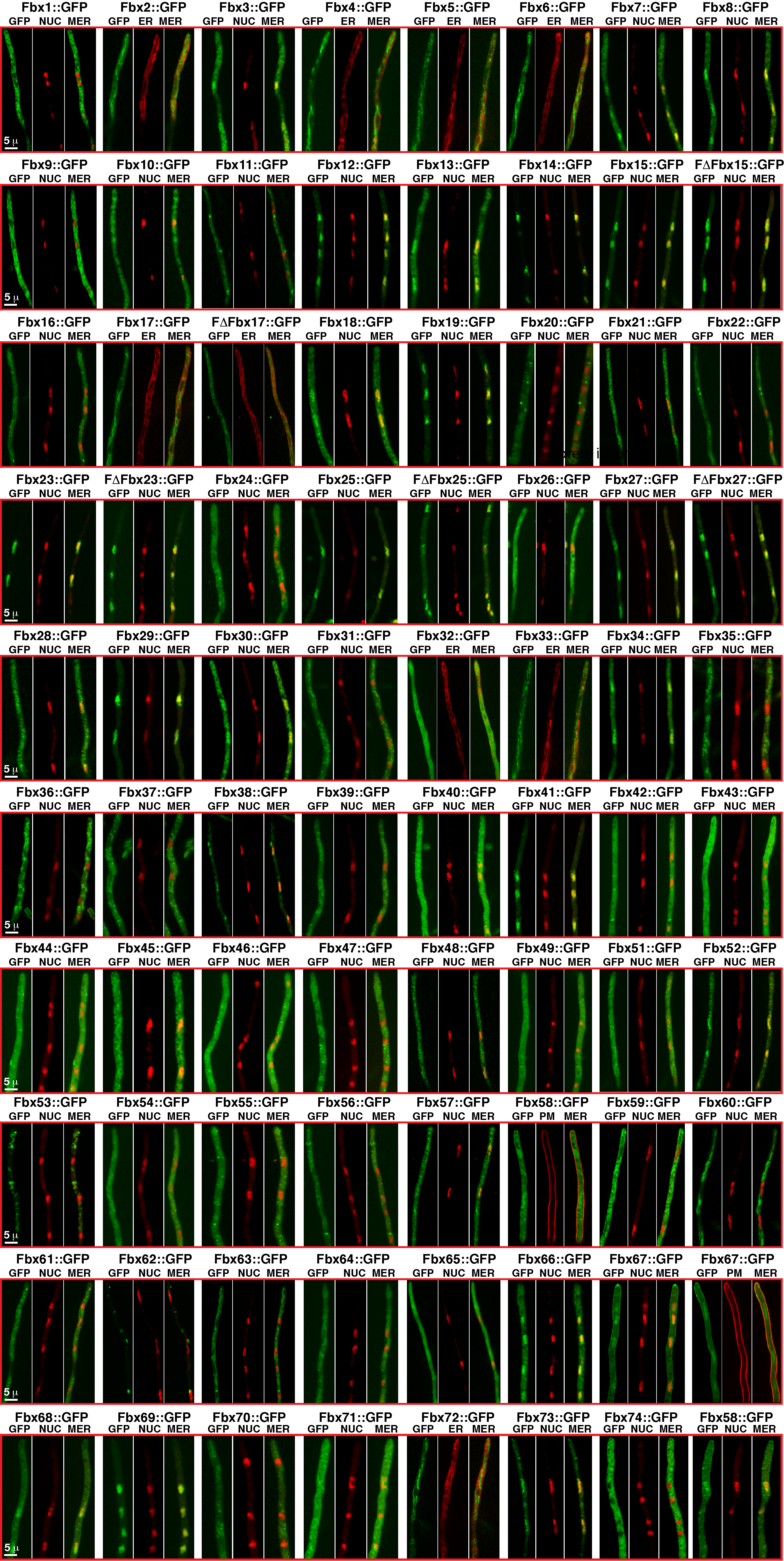

Supplement: S9 Fig — Subcellular localizations of the F-box proteins were observed under vegetative growth conditions. DRAQ5 was used to stain nuclei red, FM4-64 plasma membrane and SecA-mRFP fusion endoplasmic reticulum in red. For localization of the Fbx-GFP fusions, 400–500 spores were grown in liquid media for 16–20 hours at 30°C. Lack of F-box domains in several F-box proteins did not influence the subcellular localizations of the F-box proteins. NUC: Nucleus, MER: Merged, PM: Plasma membrane, ER: Endoplasmic reticulum. (TIF) [file pgen.1010502.s009.tif]

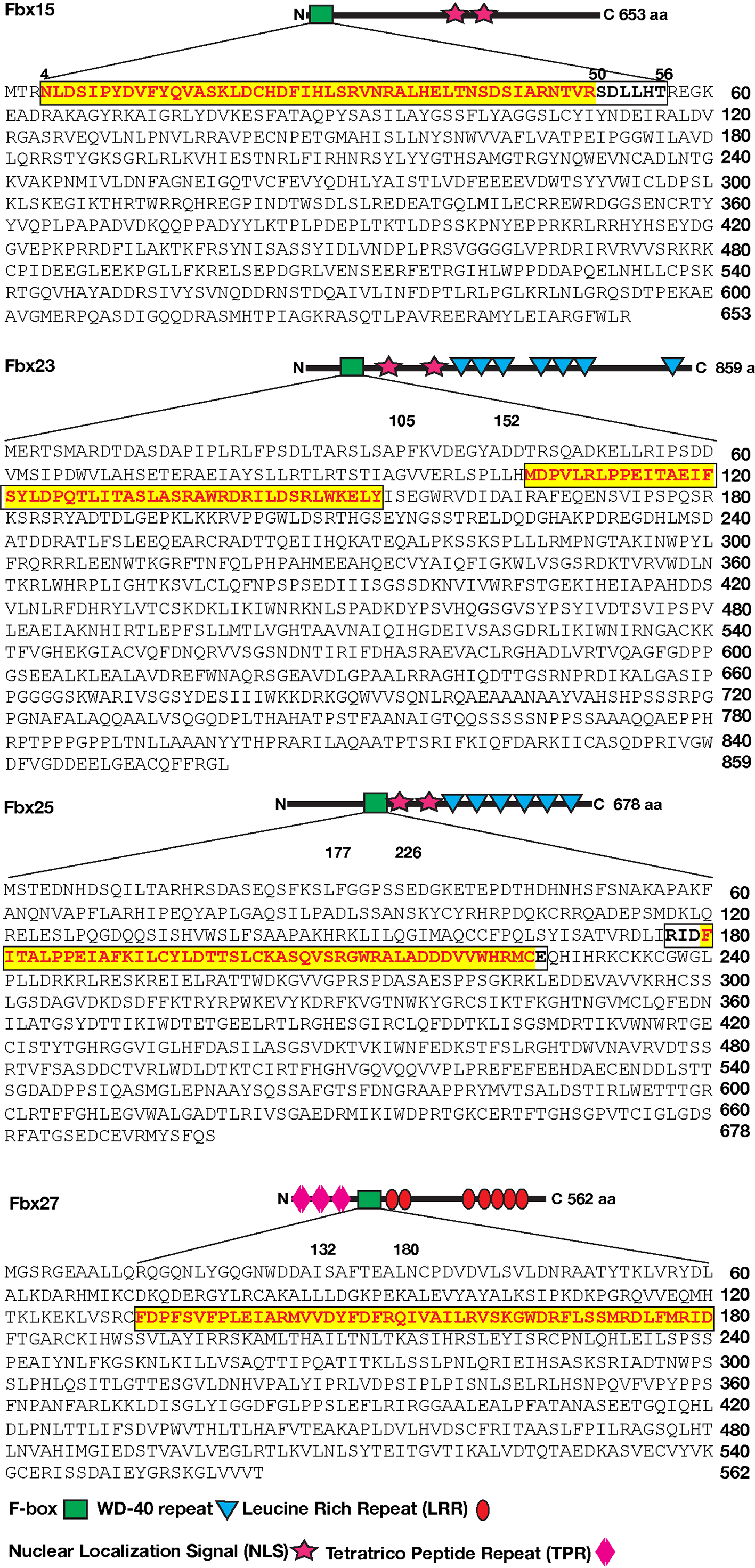

Supplement: S10 Fig — Complete amino acid sequences of Fbx15, 23, 25 and 27 are given. Squares on amino acid sequences for each protein represent the putative F-box domains. Yellow shades indicate the deleted residues. Predicted F-box domains of Fbx15 (4–50 aa), Fbx23 (105–152 aa), Fbx25 (177–226 aa), Fbx27 (132–180 aa) were deleted. (TIF) [file pgen.1010502.s010.tif]

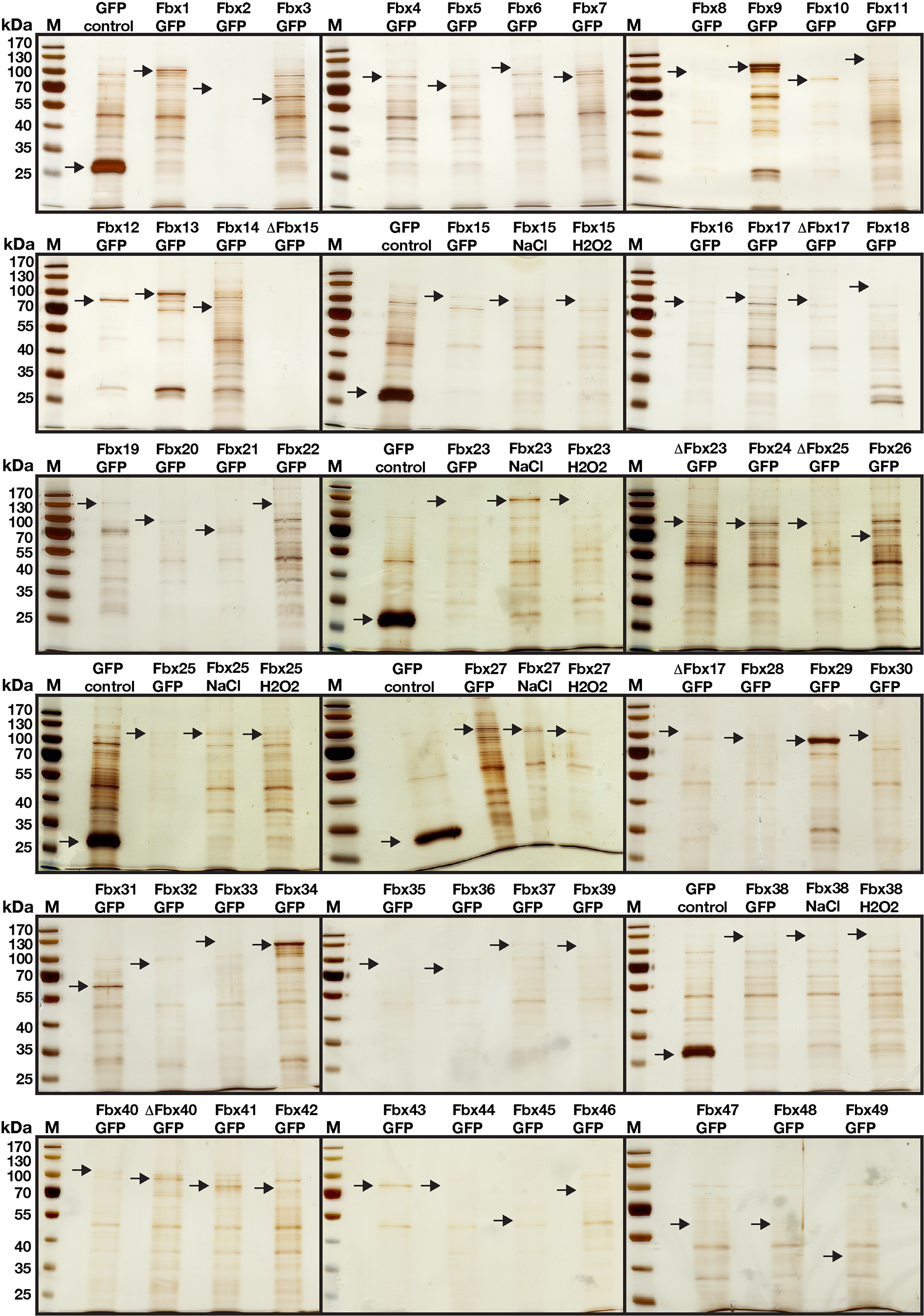

Supplement: S11 Fig — Final eluates of F-box-GFP fusions after GFP TRAP purification were run on 10% SDS polyacrylamide gel. Fat band in several gels represent only GFP control. ΔFbx15, ΔFbx17, ΔFbx23, ΔFbx25, ΔFbx27, ΔFbx40 represent GFP TRAP purifications performed without the F-box protein domains in the respective proteins. Developmentally important F-box proteins Fbx15, 23, 25, 27 and 38 (Fbx72 and 74 not shown here) were treated with two stress conditions, osmotic (NaCl) and oxidative (H2O2), respectively. Black arrows indicate expected sizes of Fbx-GFP fusion proteins. (TIF) [file pgen.1010502.s011.tif]

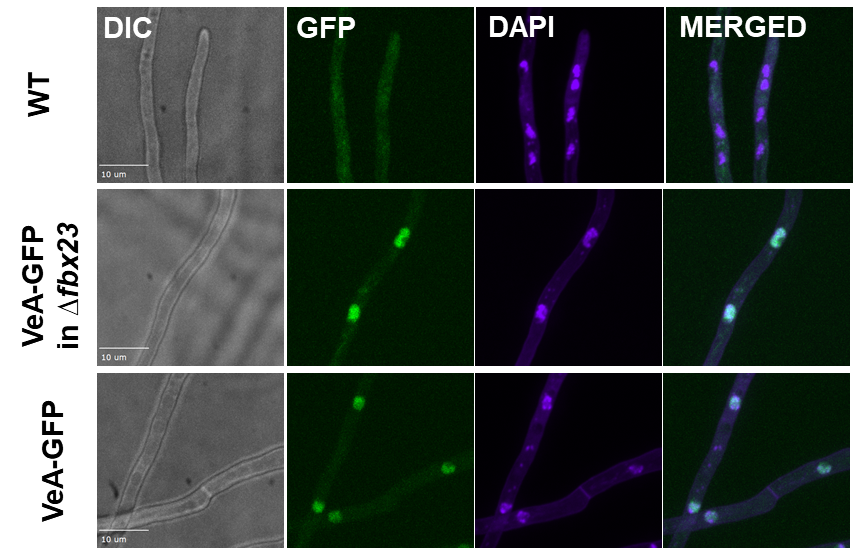

Supplement: S12 Fig — 2 000 spores were inoculated in liquid minimal medium containing microscopic chambers (Ibidi) and incubated for 18 h in light at 37°C. Microscopic pictures were taken with the Plan-Apochromat 100x/1.4 oil objective. DAPI was used to visualize nuclei. Size bars indicate 10 μm. (TIF) [file pgen.1010502.s012.tif]
