# Supplementary material for: F-box receptor mediated control of substrate stability and subcellular location organizes cellular development of Aspergillus nidulans
Source: PLoS Genet. 2022 Dec 12;18(12):e1010502. doi: 10.1371/journal.pgen.1010502 (PMC9744329; doi:10.1371/journal.pgen.1010502)
Supplement: S1 Text — (DOCX) [file pgen.1010502.s032.docx]

**S1 Text.** **Supplementary materials and methods.**

**F-box receptor mediated control of substrate stability and subcellular location organizes cellular development of *Aspergillus nidulans***

Özlem Sarikaya Bayram^1¶^, Özgür Bayram^1¶*^, Betim Karahoda^1^, Cindy Meister^2^, Anna M. Köhler^2^, Sabine Thieme^2^, Nadia Elramli^1^, Dean Frawley^1^, Jamie McGowan^1^, David A. Fitzpatrick^1^, Kerstin Schmitt^2^, Leandro Jose de Assis^3^, Oliver Valerius^2^, Gustavo H. Goldman^3^, Gerhard H. Braus^2,*^

**Nucleic acid manipulations**

Standard recombinant DNA technologies were applied for the generation of linear and circular DNA molecules. Plasmids and oligos used during this study are listed in S19 and S17 Tables, respectively. For polymerase chain reactions (PCR), Q5 Hot Start and Phusion High-Fidelity Polymerases (NEB) were used. Circular or linear DNA fragments of *fbx* deletions and *gfp* fusions were constructed as described below.

**Construction of *fbx* deletion cassettes**

Deletions and oligonucleotides are given in S16 and S17 Tables. Some of the *fbx* deletions cassettes were received from Fungal Genetics Stock Centre (FGSC), Kansas, USA. For the *fbx* deletion cassettes of FGSC origin, only three oligos were used as explained for *fbx1*. One pair (F002/139) was used for the Southern probe generations, second pair (F002/003) was used for cassette generation. Rest of the *fbx* deletion cassettes such as *fbx5* were created by amplifying 5' and 3' UTRs of respective *fbx* genes (F087/118, F088/119) from A4 genomic DNA and fusing these two fragments with an *Aspergillus fumigatus pyrG* (*AfpyrG*) marker (OZ483/484) to transform into a WT strain.

**Construction of Fbx, CulA, SkpA-GFP fusions**

Fbx-GFP plasmids are given in S18 Table. In order to construct *fbx-sgfp* fusion plasmids, following strategy was applied. First respective *fbx* open reading frame (ORF) was amplified from A4 WT genomic DNA with respective oligos such as F355/F356. The respective ORF amplicon was fused to *sgfp* (OZ908/F354) and cloned under constitutive *gpdA* promoter in *Pme*I site of pME4143 by using In-Fusion HD kit (Clontech). Plasmids were sequences in house facility Goettingen Genomics Laboratory (G2L). The constructions are given in S18 and S19 Tables. Only *fbx15*, *23*, *24*, *25* and *40* *sgfp* fusions were constructed differently. Fbx15, FΔFbx15, Fbx22, Fbx23, FΔFbx23, Fbx25, FΔFbx25, Fbx40, FΔFbx40-GFP plasmids were created as follow. Each sequenced fusion construct in *Pme*I site of pSK379 plasmid [1] were released by *Bbcv*II and cloned in *Pme*I site of pME4143 leading to respective plasmids shown in Table S19. In order to transform *fbx-sgfp* fusions into the respective *fbx* deletants, cassettes targeting the biotin locus were released by a *Swa*I digestion and 1-2 μg linear cassette was transformed for each strain. In order to construct *skpA-sgfp* fusions, *skpA* ORF including a 2 kb promoter region (BK449/450) and a terminator region (BK451/452) were amplified and fused to either *sgfp-AfpyrG* in *Sma*I site of pUC19 leading to plasmids pBK100, respectively. *culA* ORF and promoter (BK455/456) and terminator (BK457/458) were fused to *sgfp-AfpyrG* by cloning in pUC19, yielding pBK102. Cassettes carrying endogenously tagged versions of *skpA* and *culA* with *sgfp* were amplified from plasmids with nested oligos (BK453/454, for *skpA*, BK459/460 for *culA*) and transformed into WT.

**Construction of VipC-sGFP fusion**

The open reading frame (ORF) of *vipC* was amplified with oligonucleotides CM257/CM258 from genomic DNA of wild type *A. nidulans* AGB551 and resulted in a fragment of 1490 bp. Furthermore, a PreScissionProtease (PP) site, a linker and *gfp* was amplified from pME4706 with oligonucleotides CM135/CM236 and resulted in a 759 bp long fragment. Both fragments were fused through a PCR reaction using primers CM257 and CM236. The fusion product was 2249 bp long and was ligated into the *Pml*I site of pME4696 using the Seamless Cloning and Assembly Kit (Thermo Fisher Scientific) according to manufacturer´s instructions. The 961 bp long 3´flanking region was amplified with CM259 and CM260 from genomic DNA of AGB551 and ligated into the *Swa*I cutting site of the pME4696, which already contained the *vipC:gfp* in its *Pml*I restriction site. The outermost primers (CM257 and CM260 contain a *Pme*I restriction site to linearize the resulting plasmid for transformation into *A. nidulans*. The resulting plasmid was named pME4991 and the linear, with *Pme*I digested cassette was transformed into AGB551 and AGB822 resulting in AGB1286 and AGB1287, respectively.

**Construction of VeA-GFP fusion in ∆*fbx23***

AGB822 was transformed with the veA:gfp cassette resulting from *Pme*I digested pME4714 resulting in AGB1234.

**Transformations**

DH5α and MACH-1 (Invitrogen) *E. coli* strains were used for cloning [2]. Fungal or bacterial transformation were described [3].

**Immunoblotting**

Detection of SkpA and GFP proteins from 80 µg protein extracts were performed as described [4] using monoclonal GFP (Santa Cruz, sc-9996) or custom made polyclonal α-SkpA (GenScript) antibodies. For VipC-GFP fusion, 100 μg of proteins were loaded on a 12 % SDS-PAGE and separated by size. α-GFP antibody was used in 1:500 dilution in 5 % (w/v) TBS-T milk powder solution. As secondary antibody α-mouse was applied to the membrane in a 1:1000 dilution in 5 % (w/v) TBS-T milk powder. Detection of chemiluminescent signals and quantification of signal intensities was performed as described previously [5].

**Hybridization methods**

DNA/DNA and RNA/DNA hybridizations were performed as explained in detail earlier [6].

**High-performance liquid chromatography (HPLC) analysis of secondary metabolites**

5x10^3^ spores of WT and deletion strains were inoculated on GMM agar supplemented with vitamins and 1% Oatmeal at 37$^{\circ}$C for 5 days. A sample was cut out from the centre of the plate with the back of a 10 ml falcon tube and cut into pieces. 3 ml of ddH_2_O was added to diced samples and vortexed for 5 min on ice followed by addition of 3 ml chloroform. Samples were vortexed for a further 5 min and shaken on a rotator at max speed at 4$^{\circ}$C for 30 minutes. Extracted samples with chloroform were centrifuged at 5000 rpm at 4 $^{\circ}$C for 15 min. Lower phase was removed into a new vial and dried in a speed vac at medium heat for 20-30 minutes. HPLC analysis was performed on a Shimadzu RP-HPLC with a photodiode array detector (PDA). Sterigmatocystin (Sigma) 1mg/ml was used as the standard (2.5 μl of standard added in 47.5 μl of 100% Methanol). The samples were resuspended in 200 μl Methanol and injected onto a Luna Omega 5 μm polar C18 (LC column 150 x 4.6m.m) and separated across at water: acetonitrile gradient with 0.1% (v/v) TriFluoroacetic Acid (TFA).

**Protein crude extract preparation and Western Blot**

A. nidulans cultures were inoculated with 1X10^6^ spores/ml and grown for 20 h in light under agitation at 37°C in minimal medium. Mycelia was harvested after vegetative growth and transferred on solid agar plates for the induction of asexual or sexual development. Samples for protein isolation were taken after vegetative growth and after 8, 16 and 24 hours after induction of multicellular development. Samples were frozen in liquid nitrogen and manually grained. Around 300 μl of grained mycelia was used for protein extraction. Therefore, buffer B* (300 mM NaCl, 100 mM Tris pH 7.5, 10 % (v/v) glycerol, 0.5 mM EDTA, 0.05% (v/v) NP-40) was freshly supplemented with 1.5 mM DTT, 1 mM PMSF and 1 tablet/50 ml complete EDTA-free protease inhibitor cocktail (Roche). Proteins were extracted like described in [5].

**GFP trap protocol and LC-MS protein identification**

Immunoprecipitation (IP) of SkpA, CulA and 49 Fbx-GFP fusions [2] employed GFP-TRAP sepharose (Chromotek). 5-7 ml pulverized mycelia were lysed and vortexed on ice by addition of 5 ml breaking buffer B [200 mM NaCl, 50 mM Tris pH 7.5, 1 mM EDTA, 0.1% (v/v) NP-40, 1.5 mM DTT, 10% Glycerol, 1.25 mM Benzamidine, 1 mM PMSF, 1x Phosphatase inhibitor cocktail pill and 2x complete EDTA free protease inhibitor pills (Roche) (for 100 ml B buffer)]. Mixture was centrifuged at 4500 rpm for 30 min at 4°C and the supernatant was transferred into a new precooled 10 ml falcon tube. 15 μl GFP-TRAP sepharose beads were washed in 250 μl B buffer and spun down at 1000 rpm for 1 min at 4°C. The beads were added to the supernatant and incubated on the rotator for 2 h at 4°C. After incubation, the beads were spun down at 2500 rpm 1 min at 4°C. The beads were washed once with 5 ml and twice with 1.5 ml B buffer and spun down at 4000 rpm for 1 min at 4°C. The remaining buffer was removed and beads were resuspended in 3X loading dye by boiling for 6-10 min. Samples were run on a 10% SDS polyacrylamide and stained with silver followed by in-gel trypsin digestion performed as described [7,8].

IP of some GFP fused Fbx proteins were performed with GFP-TRAP magnetic particles (Chromotek). 2 ml pulverized frozen fungal mycelia were mixed with 1 ml B buffer and vortexed. Mixture was centrifuged at 13000 rpm at 4°C for 10 min. Supernatant was transferred into a new 1.5 ml vial. 20 μl GFP-TRAP magnetic beads were washed with 200 μl B buffer and added to protein extracts from Fbx-GFP fusions. Beads in protein extracts were incubated on the rotator for 2 h at 4°C and separated from protein extract and washed with 1 ml B buffer three times. For final wash step, no protease inhibitor was included in B buffer. Proteins bound to magnetic beads were digested directly on the beads by addition of protease max and trypsin as given in detail followed by C-18 zip tip purification procedure before running in LC-MS [9]. LC-MS identifications of Fbx-GFP fusions were carried out by Proteome Discoverer Version 1.4 (Thermo Fisher). Specific Fbx interaction partners were determined by substracting proteins found only in GFP control.

**Mass spectrometry analysis with nanoLC-nanoESI-MS/MS2**

Nano LC - *RSLCnano Ultimate 3000* system (Thermo Scientific):

Peptides of 1-6 µl sample solution were trapped and washed on an *Acclaim® PepMap 100* column (75 µm x 2 cm, C18, 3 µm, 100 $Å$, P/N164535 Thermo Scientific) at a flow rate of 4 µl/min for 12 min. Analytical peptide separation by reverse phase chromatography was done on an *Acclaim® PepMap RSLC* column (75 µm x 15 cm, C18, 3 µm, 100 $Å$, P/N164534 Thermo Scientific) running a gradient from 96% solvent A (water, 0.1% formic acid) and 4% solvent B (80% acetonitrile, 20% water, 0.1% formic acid) to 50% solvent B within 25 min at a flow rate of 300µll/min (Fisher Chemicals).

Nano ESI mass spectrometry - *Orbitrap Velos Pro* (Thermo Scientific):

Chromatographically eluting peptides were on-line ionized by nano-electrospray (nESI) using the *Nanospray Flex Ion Source* (Thermo Scientific) at 2.4 kV and continuously transferred into the mass spectrometer. Full scans within the mass range of 300-1850 amu were collected from the Orbitrap-FT analyzer at a resolution of 30.000 (using m/z 445.120025 as lock mass) with parallel data-dependent top 10 MS2-fragmentation within the *LTQ Veleo Pro* linear ion trap. LCMS method programming and data acquisition was done with the software *XCalibur 2.2* (Thermo Fisher) and method/raw data validation with the program *RawMeat 2.1* (Vast Scientific). MS/MS2 data processing for protein analysis and identification was done with the *Proteome Discoverer 1.3* (*PD*, Thermo Scientific) and the *Discoverer Deamon 1.3* (Thermo Scientific) softwares using the Sequest (and/or Mascot) peptide analysis algorithm(s) and organism-specific taxon-defined protein databases extended for the most common contaminants. Details concerning the Mass spectrometer tuning/calibration can be found in Table A of S1 Data, the LCMS method in Table B of S1 Data, and furthermore the Settings/filters for *PD* data analysis in Table C in S1 Data.

**Generation of Fbx interactome networks**

Fbx interactome networks were visualised and annotated using Gephi [10]. Each node represents a protein. Detected protein interactions are represented by edges joining proteins. F-box proteins are highlighted.

**Ortholog analysis in model organisms**

Orthologs of *A. nidulans* Fbx proteins and their interacting proteins involved in the regulation of biological processes were identified in model organisms using a reciprocal best BLAST hits strategy. An all-versus-all BLASTp search [11] was run against *A. nidulans* and each model organism with an E-value cut-off of 10^-10^. Orthologs are defined as a pair of proteins that are each other’s best BLAST hit.

**Confocal microscopy**

For Fbx-GFP localizations, nuclei were stained in red by incubating cells in the presence of 5 μM DRAQ5 dye (Abcam) for 5-10 minutes. Plasma membrane were visualized by incubating cells in the presence of 1 μM FM4-64 (Thermo Fisher) for 5-10 minutes. For VipC-GFP and VeA-GFP localizations, 1.5-3X10^4^ spores were inoculated in 400 μl minimal medium that was placed in sterile cover slides into a sterile petri dish. Spores were distributed evenly in the solution by pipetting carefully up and down. *A. nidulans* strains were grown for 18 h at 37°C and in light. Afterwards, the remaining media was removed with a tissue and the cover slide was placed upside down on an object slide, where 20 μl fresh minimal medium were placed before. The cover slide was fixed with nail polish and used for microscopy. DAPI (4´,6-diamidino-2-phenylindole) staining was used for visualizing nuclei in *A. nidulans* hyphae.

For confocal microscopy the Zeiss AxioObserver Z.1 inverted confocal microscope with the 100x/1.4 oil objective and a QuantEM:512SC camera (Photometrics) was used. The SlideBook 6.0 software package (Intelligent Imaging Innovations) was used for taking and adjusting pictures. The quantification of signal intensities was performed with samples that were not stained with DAPI. The background intensity was substracted from the intensity values of hyphae or nucleus. Different regions in the hyphae were selected for quantification, whereby each cytoplasmic region was assigned to a single nucleus and a ratio between both was calculated. The regions used for quantification of signal intensities were selected manually. Due to a lack of predominant nuclear accumulation of VipC-GFP in the wild type we assumed that half of the selected regions were nuclei and the other half cytoplasmic regions. For each strain the two highest and the two lowest ratios were not considered for the calculation.

**SUPPLEMENTARY REFERENCES**

1. Szewczyk E, Krappmann S. Conserved regulators of mating are essential for Aspergillus fumigatus cleistothecium formation. Eukaryot Cell. 2010;9: 774–83. doi:10.1128/EC.00375-09

2. Bayram Ö, Bayram ÖS, Ahmed YL, Maruyama J, Valerius O, Rizzoli SO, et al. The *Aspergillus nidulans* MAPK module AnSte11-Ste50-Ste7-Fus3 controls development and secondary metabolism. Madhani HD, editor. PLoS Genet. 2012;8: e1002816. doi:10.1371/journal.pgen.1002816

3. Punt PJ, van den Hondel CAMJJ. [39] Transformation of filamentous fungi based on hygromycin b and phleomycin resistance markers. In: Wu R, editor. Methods in Enzymology. Cambridge, Massachusetts: Academic Press; 1992. pp. 447–457. doi:10.1016/0076-6879(92)16041-H

4. Sarikaya-Bayram Ö, Bayram Ö, Feussner K, Kim J-H, Kim H-S, Kaever A, et al. Membrane-Bound Methyltransferase Complex VapA-VipC-VapB Guides Epigenetic Control of Fungal Development. Dev Cell. 2014;29: 406–420. doi:10.1016/j.devcel.2014.03.020

5. Meister C, Thieme KG, Thieme S, Köhler AM, Schmitt K, Valerius O, et al. COP9 Signalosome Interaction with UspA/Usp15 Deubiquitinase Controls VeA-Mediated Fungal Multicellular Development. Biomolecules. 2019;9: 238. doi:10.3390/biom9060238

6. Christmann M, Schmaler T, Gordon C, Huang X, Bayram O, Schinke J, et al. Control of multicellular development by the physically interacting deneddylases DEN1/DenA and COP9 signalosome. Heitman J, editor. PLoS Genet. 2013;9: e1003275. doi:10.1371/journal.pgen.1003275

7. Bayram Ö, Krappmann S, Ni M, Bok JW, Helmstaedt K, Valerius O, et al. VelB/VeA/LaeA complex coordinates light signal with fungal development and secondary metabolism. Science. 2008;320: 1504–6. doi:10.1126/science.1155888

8. von Zeska Kress MR, Harting R, Bayram Ö, Christmann M, Irmer H, Valerius O, et al. The COP9 signalosome counteracts the accumulation of cullin SCF ubiquitin E3 RING ligases during fungal development. Mol Microbiol. 2012;83: 1162–1177. doi:10.1111/j.1365-2958.2012.07999.x

9. O’Keeffe G, Hammel S, Owens RA, Keane TM, Fitzpatrick DA, Jones GW, et al. RNA-seq reveals the pan-transcriptomic impact of attenuating the gliotoxin self-protection mechanism in Aspergillus fumigatus. BMC Genomics. 2014;15: 894. doi:10.1186/1471-2164-15-894

10. Bastian M, Heymann S, Jacomy M. Gephi: An Open Source Software for Exploring and Manipulating Networks. Proc Int AAAI Conf Web Soc Media. 2009;3: 361–362. Available: https://ojs.aaai.org/index.php/ICWSM/article/view/13937/13786

11. Altschul SF, Madden TL, Schäffer AA, Zhang J, Zhang Z, Miller W, et al. Gapped BLAST and PSI-BLAST: a new generation of protein database search programs. Nucleic Acids Res. 1997;25: 3389–402. doi:10.1093/nar/25.17.3389
